# Mitochondria-targeting natural product rhein conjugated with dichloroacetate as the dual inhibitor of glycolysis and oxidative phosphorylation to off energize cancer cells and induce ROS storm

**DOI:** 10.7150/thno.107812

**Published:** 2025-03-31

**Authors:** Zhili Zhang, Shiming Tang, Minghui Qi, Hongyang Zhao, Meng Wu, Shi-Wen Huang

**Affiliations:** 1 Key Laboratory of Biomedical Polymers of Ministry of Education, Department of Chemistry, Wuhan University, Wuhan 430072, P. R. China.; 2 Department of Ultrasound, Zhongnan Hospital of Wuhan University, Wuhan University, Wuhan 430071, P. R. China.

**Keywords:** glycolysis, reactive oxygen species, mitochondria-targeting, oxidative phosphorylation, immunogenic cell death

## Abstract

**Rationale:** Metabolic reprogramming emerges as a remarkable hallmark of cancer cells and exhibits potential in the development of metabolic modulators. Numerous small-molecule inhibitors mainly target reversing the dominant-glycolysis pathway. However, energy metabolic adaptation that facilitates the alternation of metabolic phenotypes from glycolysis to oxidative phosphorylation (OXPHOS) undermines treatment efficacy. Thus, small molecular therapeutic agents, concurrently cutting off the cellular energy metabolism of glycolysis and OXPHOS and trigger oxidative stress damage, hold promise for cancer therapy.

**Methods:** Herein, natural product rhein with the capacity of mitochondria-targeting was conjugated with pyruvate dehydrogenase kinase (PDK) inhibitor dichloroacetate (DCA) to form a multifunction small molecule drug Rhein-DCA conjugate. The ATP production inhibition, oxidative stress damage and antitumor efficacy of Rhein-DCA conjugate were evaluated both *in vitro* and *in vivo*.

**Results:** Rhein unit not only led to the effective accumulation of Rhein-DCA conjugate in mitochondria, but also promoted the binding of DCA and PDK1, enhancing typical inhibition of glycolysis by DCA *via* PDK-PDH axis. Unlike classical PDK inhibitors, which restrained glycolysis and restored OXPHOS, rhein within the conjugate further suppressed mitochondrial respiratory chain complex and induced sustained opening of mitochondrial permeability transition pore, destroying intractable OXPHOS. Importantly, rhein component in the conjugate elevated the reactive oxygen species (ROS) level to further disrupt OXPHOS, and thus ROS triggered the release of damage associated molecular patterns. Simultaneously, the conjugate weakened lactate-mediated immunosuppression by reducing lactate levels in the tumor microenvironment. Eventually, the polarization state of tumor-associated macrophages could be effectively reversed following oral administration.

**Conclusion:** This study designed a small-molecule dual-inhibitor of glycolysis and OXPHOS to circumvent metabolic adaptations and simultaneously induce immunogenic cell death for macrophages repolarization, thereby synergistically promoting antitumor efficacy.

## Introduction

Energy metabolism reprogramming is a crucial hallmark of cancers, playing a vital role in tumor progression and invasion 1, 2. Unlike normal cells, the predominate energy source of cancer cells is elevated aerobic glycolysis rather than typical mitochondrial oxidative phosphorylation (OXPHOS), which is an adaptation to the adverse nutrient-deficient caused by rapid proliferation, known as "Warburg effect" 3, 4. This unique glucose metabolism has emerged as a promising target for cancer treatment. Numerous small-molecule enzymes or kinases inhibitors have been developed, focusing on targeting and restraining the energy synthesis of strong glycolysis, restoring OXPHOS of cancer cells at the initiation of treatment, and ultimately driving tumor to suicide by apoptosis 5-7. Unfortunately, mitochondria are not defective in most cancer cell, energy metabolic adaptability would enable tumor cells to resume survival by obtaining adenosine triphosphate (ATP) from the sub-dominant OXPHOS, thereby compensating for the energy deficiency of glycolytic blockade 8-11. Apparently, drugs that only blocking glycolysis turn to be not feasible for treatment. On the other side, small molecular therapeutic drugs recently, concentrating on mitochondrial OXPHOS, have also achieved positive results in anti-tumor aspect 2, 12. Most metabolic modulators focus on regulating a single energy metabolic pathway, such as metformin (an AMP-activated protein kinase activator that inhibits mitochondrial complex I) and 2-deoxy-D-glucose (2-DG) (a glycolysis inhibitor), but their efficacy is still limited by tumor metabolic plasticity 7, 13. These have inspired a number of trials aimed at evading metabolic adaptability of cancer cells by drug combination of individual glycolysis and OXPHOS inhibitors to jointly dismantle glycolysis and OXPHOS for effective cancer therapy 14-16. However, glycolysis is a cytoplasmic-based metabolic process, OXPHOS is centered in the mitochondria, the inherent spatiotemporal differences in the mechanisms make a single small molecule drug with dual-inhibition of glycolysis and OXPHOS is challenging.

The highly expressed pyruvate dehydrogenase kinases (PDK) in the mitochondria have turned to be a hotspot target due to its important role in increasing glycolysis in many kinds of tumor cells 17. The development of PDK inhibitors has also become a potential therapeutic approach for cancer treatment. Dichloroacetate (DCA) is the most extensively investigated PDK inhibitor to alter the aberrant energy metabolism reprogramming in multiple carcinomas and is currently in the stage of clinical trial 7. DCA binds the overexpressed PDK within the mitochondria of cancer cells and reactivates the pivotal enzyme pyruvate dehydrogenase complex (PDH), thereby redirecting pyruvate towards tricarboxylic acid cycle (TCA) instead of glycolysis, and resulting in the energy metabolic shift from glycolysis to unfavorable OXPHOS of cancer cells and provoking tumor apoptosis 18-20. Due to low cellular uptake of DCA and poor mitochondrial location after entrancing cancer cells, high dose of DCA is required for efficient cancer therapy, which limits the extensive application of DCA in cancer treatments 21.

Rhein, 4,5-dihydroxyanthraquinone-2-carboxylic acid, is a natural compound possessing mitochondrial-targeting antitumor activity 8, 22. Previous studies indicated that rhein can bind to a variety of molecules on the mitochondrial membrane to cause the pathological opening of molecular transport gated mitochondrial permeability transition pore (mPTP) to directly boost the mitochondrial permeability, eventuate the loss of mitochondrial membrane potential (Δψm) and the uncoupling of OXPHOS in various carcinomas 23-25. Moreover, rhein also disrupt mitochondrial respiratory chain to evoke excessive reactive oxygen species (ROS) in cancer cells, which further damages the cancer cell mitochondria and impairs subsequent ATP synthesis through OXPHOS 25, 26. In contrast to the non-specific ROS-inducing agents, such as arsenic trioxide (ATO), which suffers severe systemic toxicity at high doses, the mitochondria-targeting compound would minimize off-target effects 27.

**Scheme 1:**
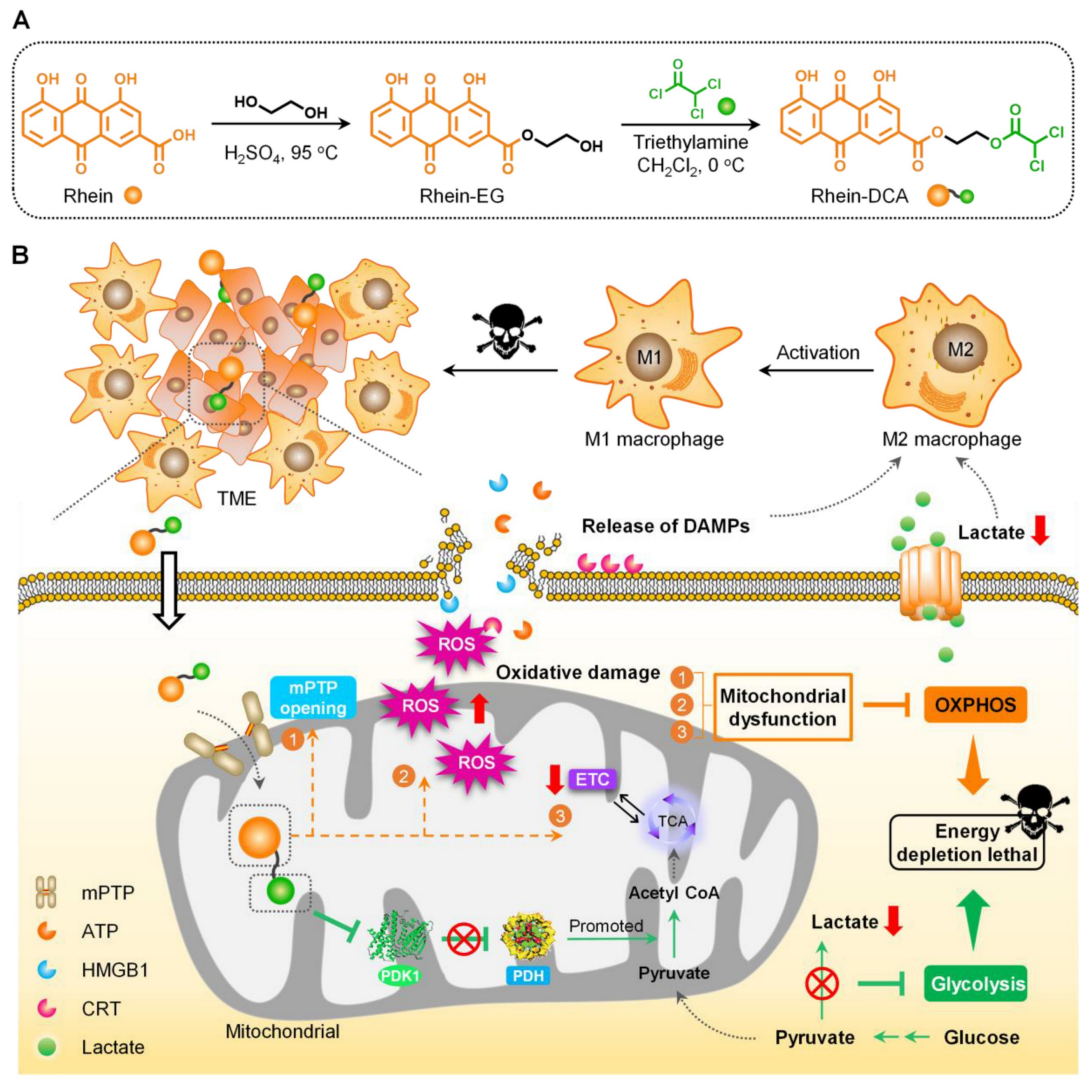
Design and synthesis of mitochondrial-targeting Rhein-DCA conjugate for off energizing cancer cells by inhibiting glycolysis and OXPHOS, and re-polarizing M2 TAMs into the M1 phenotype for synergistic tumor therapy. A) Illustration of the preparation processes of Rhein-DCA conjugate with reaction conditions and reagents. B) Schematic demonstrated that Rhein-DCA conjugate simultaneously cuts off energy supply of glycolysis and OXPHOS by PDK-PDH axis and mitochondrial dysfunction. Meanwhile, the increasing intracellular ROS further impedes OXPHOX and triggers the emission of DAMPs for macrophage repolarization to achieve synergistic tumor treatment.

In this work, we rationally design and synthesize the mitochondrial-targeting Rhein-DCA conjugate and evaluate its antitumor mechanism. In this conjugate, DCA unit acts as the component for PDK inhibition and contributes to glycolysis inhibition, while rhein in the conjugate not only functions as a mitochondrial-targeting ligand, but also contributes to OXPHOS inhibition. Rhein-DCA conjugate acts as the oral small molecule dual-inhibitor of glycolysis and OXPHOS to impede cellular energy production and prompt cancer cells death (Scheme 1). Besides the important mitochondrial-targeting role of rhein in Rhein-DCA conjugate, we also found the introduction of rhein can significantly increase the ability of DCA to bind PDK1 and down-regulate the expression of PDK1 gene. The potent inhibition of PDK1 with Rhein-DCA in the mitochondria rescues PDH complex for initiating TCA cycle, and inhibiting glycolysis energy supply and consequent accumulation of lactate in tumor microenvironment (TME). Meanwhile, OXPHOS, the main adaptation metabolism in PDK inhibitor-treated cancer cells, was also inhibited with rhein in Rhein-DCA conjugate to prevent tumor cells from obtaining ATP *via* alternative OXPHOS metabolism pathway. As a result, the dual inhibition of glycolysis and OXPHOS strategy of Rhein-DCA conjugate achieved remarkable anticancer effect. The synergistic effects of Rhein-DCA are far superior to the mere combination of rhein and DCA. More importantly, the amplified intracellular ROS storm in the 4T1 cells treated with Rhein-DCA conjugate induced the emission of immunostimulatory damage-associated molecular patterns (DAMPs) which combined with lactate decline, re-polarizing M2-tumor-associated macrophages (TAMs) into M1 phenotype in the TME, which is also beneficial to cancer therapy *in vivo*.

## Results and Discussion

### Design and characterization of Rhein-DCA conjugate

The small molecular inhibitor Rhein-DCA conjugate for off energizing of tumor cells consists of a mitochondria-targeting rhein with OXPHOS-disturbing capability and a glycolytic inhibitor DCA fragment, with ethylene glycol (EG) as the linker. Firstly, intermediate Rhein-EG was prepared from EG substrate and rhein through the esterification catalyzed by sulfuric acid in 81.8% yield, then the DCA moiety was introduced to Rhein-EG by further esterification with dichloroacetyl chloride, obtaining the Rhein-DCA conjugate (Scheme 1A). The formation and chemical structure of Rhein-EG and Rhein-DCA conjugate were confirmed with ^1^H nuclear magnetic resonance (^1^H NMR), ^13^C nuclear magnetic resonance (^13^C NMR) spectroscopy, high resolution mass spectroscopy (HRMS) and fourier transform infrared spectrometer (FT-IR) (Figures S1-S7). What's more, the absorption and emission properties of Rhein-DCA conjugate indicated its potential in biological imaging (Figure S8A-C). More detailed synthesis and characterizations were illustrated in the supporting information.

### Stability of Rhein-DCA conjugate

Stability of drugs is vital indexes for anticancer application. The stability of Rhein-DCA conjugate *in vitro* was performed by dispersing it in 2.5% dimethyl sulfoxide (DMSO)-PBS at room temperature for 0, 2, 6 and 12 h. UV-Vis absorption spectral results showed that minor changes of configuration and intensity within 12 h (Figure S9A), indicating that the Rhein-DCA conjugate is relatively stable. Furthermore, we also quantitatively analyzed the intracellular stability of Rhein-DCA using high-performance liquid chromatography (HPLC). After 4 h of uptake, the intracellular concentration of Rhein-DCA was 0.095 μg/10^6^ cells within 4T1 cells. Upon removing the Rhein-DCA in the media and incubating for another 2 h, the residual Rhein-DCA concentration within 4T1 cells remained at 0.080 μg/10^6^ cells, corresponding to a degradation rate of approximately 15.7% within 2 h. This indicated that Rhein-DCA predominantly remained intact within tumor cells, with a minor part might being cleaved or excreted (Figure S9B-C).

### Cellular uptake and cytotoxicity assay *in vitro*

**Figure 1:**
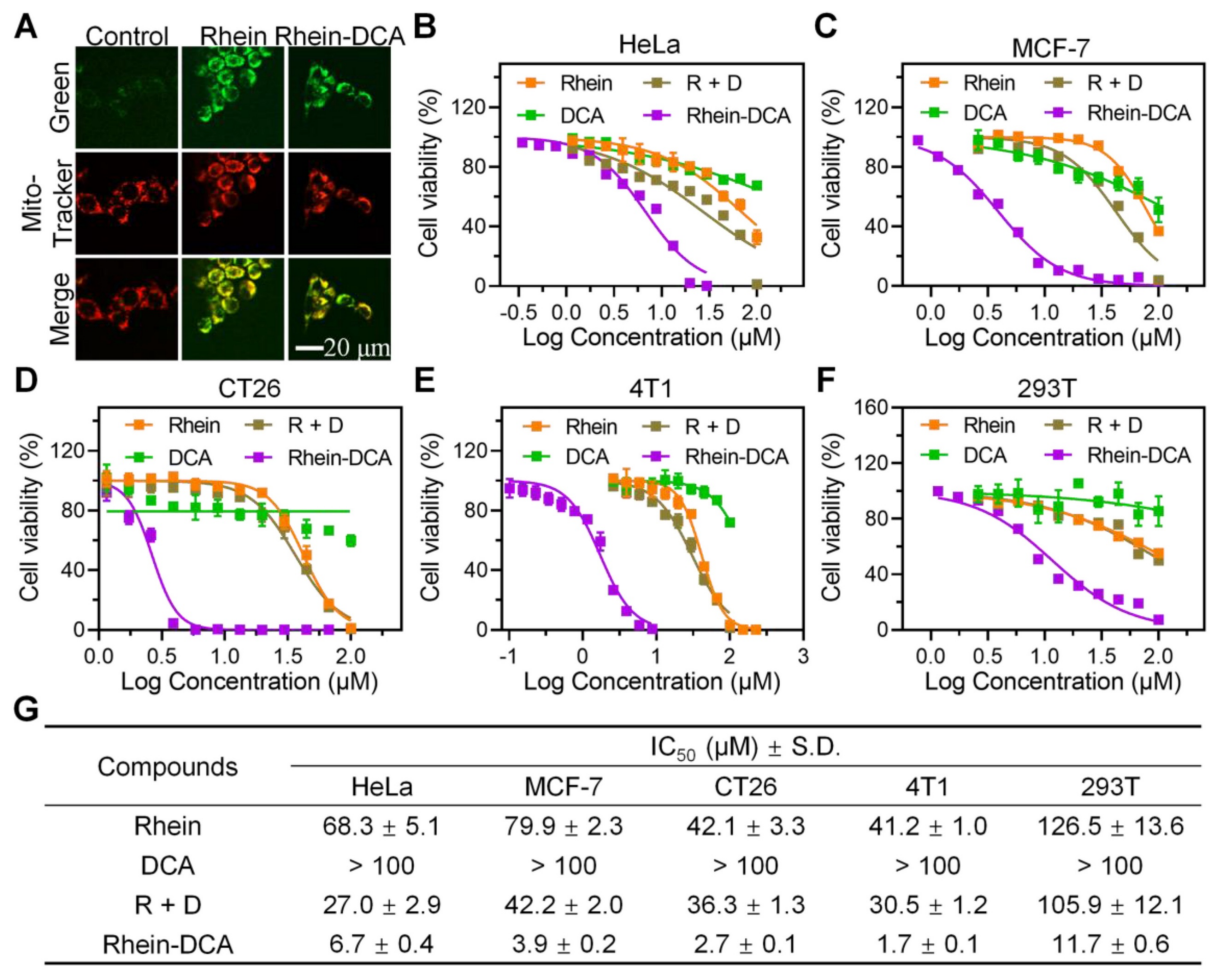
Cytotoxicity of Rhein-DCA conjugate to various cancer cell lines. A) Confocal microscopy images of 4T1 cells treated with rhein and Rhein-DCA and then stained MitoTracker Deep Red FM. Scale bar represents 20 μm. B), C), D), E) and F) Relative cell viabilities of Hela, MCF-7, CT26, 4T1 and 293T cells treated by DCA, rhein, R + D and Rhein-DCA conjugate for 72 h. G) IC50 values [μM] of rhein, DCA, R + D and Rhein-DCA conjugate towards HeLa, MCF-7, CT26, 4T1 and 293T cell lines after 72 h treatment. Data represent mean ± S.D., n = 3.

In order to evaluate the positive effect of the conjugation of rhein and DCA on anti-cancer aspect, cellular uptake of Rhein-DCA conjugate in 4T1 cells was first examined by confocal laser scanning microscope (CLSM). Based on the fluorescence properties of Rhein-DCA conjugate, green fluorescence signal indicated its distribution within the cells. As shown in Figure S10, the green fluorescence intensity within cytoplasm gradually increased with the extension of co-incubation time, demonstrating that Rhein-DCA conjugate could effectively penetrate cell membrane. To further confirm the mitochondrial targeting ability of Rhein-DCA conjugate due to the coupling of rhein, 4T1 cells were stained with Mito Tracker Deep Red FM, after being treated with rhein and Rhein-DCA conjugate for 24 h, and the CLSM images showed in Figure 1A. The green fluorescence of rhein was highly overlapped with the red fluorescence of mitochondria (Mito tracker red) and merged into yellow fluorescence in rhein and Rhein-DCA groups. Correspondingly, pearson's correlation coefficient of rhein and Rhein-DCA conjugate in Figure 1A were also calculated to be 0.91 and 0.92 by ImageJ, respectively, suggesting that the preeminent mitochondrial targeting ability of Rhein-DCA conjugate. In addition, the green fluorescence of Rhein-DCA almost did not co-localize with the red fluorescence of lysosomes (Lyso tracker red) and endoplasmic reticulum (ER tracker red), demonstrating the inefficient targeting toward these organelles (Figure S11).

The cytotoxicity of Rhein-DCA conjugate was performed on various cancer cell lines, including breast cancer (4T1 and MCF-7), cervical cancer (HeLa), and colorectal carcinoma (CT26) *via* 3-(4,5)-dimethylthiahiazo(-z-y1)-3,5-di-phenytetrazoliumromide (MTT) assay. The cytotoxicity and corresponding half maximal inhibitory concentration (IC_50_) were showed in Figure 1B-E and Figure 1G. Similar to previous reports, single rhein showed low cytotoxicity with IC_50_ value ranging approximately from 40 to 80 μM in all tested cells lines, and lower toxicity with IC_50_ > 100 μM was observed in different cell lines after single DCA treatment due to its adverse physicochemical properties, while the toxicity of their combination (designated as R + D) was only slightly increased. Encouragingly, in comparison with individual rhein, DCA, and their combination, the conjugation of rhein and DCA significantly increased cytotoxicity by at least about 10-fold in all four cancer cell lines, demonstrating the beneficial role of rhein in promoting the abundant mitochondrial accumulation of DCA. Moreover, the cytotoxicity of these compounds (rhein, DCA, R + D and Rhein-DCA) for normal cells (293T) was lower than that of those tested cancer cells. The IC_50_ value of Rhein-DCA conjugate for 293T cells was 11.7 μM, which is approximately 2~8 fold lower than that of cancer cells, suggesting a relatively mild side-effect profile (Figure 1F).

Moreover, by comparing the IC_50_ value of above cancer cells after Rhein-DCA treatment, we noticed that Rhein-DCA conjugate showed lower cytotoxicity (IC_50_ 6.7 μM) against HeLa cells, while exhibiting higher cytotoxicity towards MCF-7 (IC_50_ 3.9 μM), CT26 (IC_50_ 2.6 μM), and particularly 4T1 (IC_50_ 1.7 μM) cell lines (Figure 1G). The disparities in cytotoxicity could be attributed to the distinct metabolic profiles of these cancer cell lines. HeLa exhibited poor glycolysis and had low expression of PDK1, while MCF-7, CT26 and especially 4T1 manifested high glycolytic level along with the overexpression of PDK1 28. Hence, more studies were subsequently carried on 4T1 cells to better investigate the regulatory effect of Rhein-DCA conjugate on PDK1 related to metabolism.

### Effects of Rhein-DCA conjugate on glycolytic pathway and whole genome

**Figure 2:**
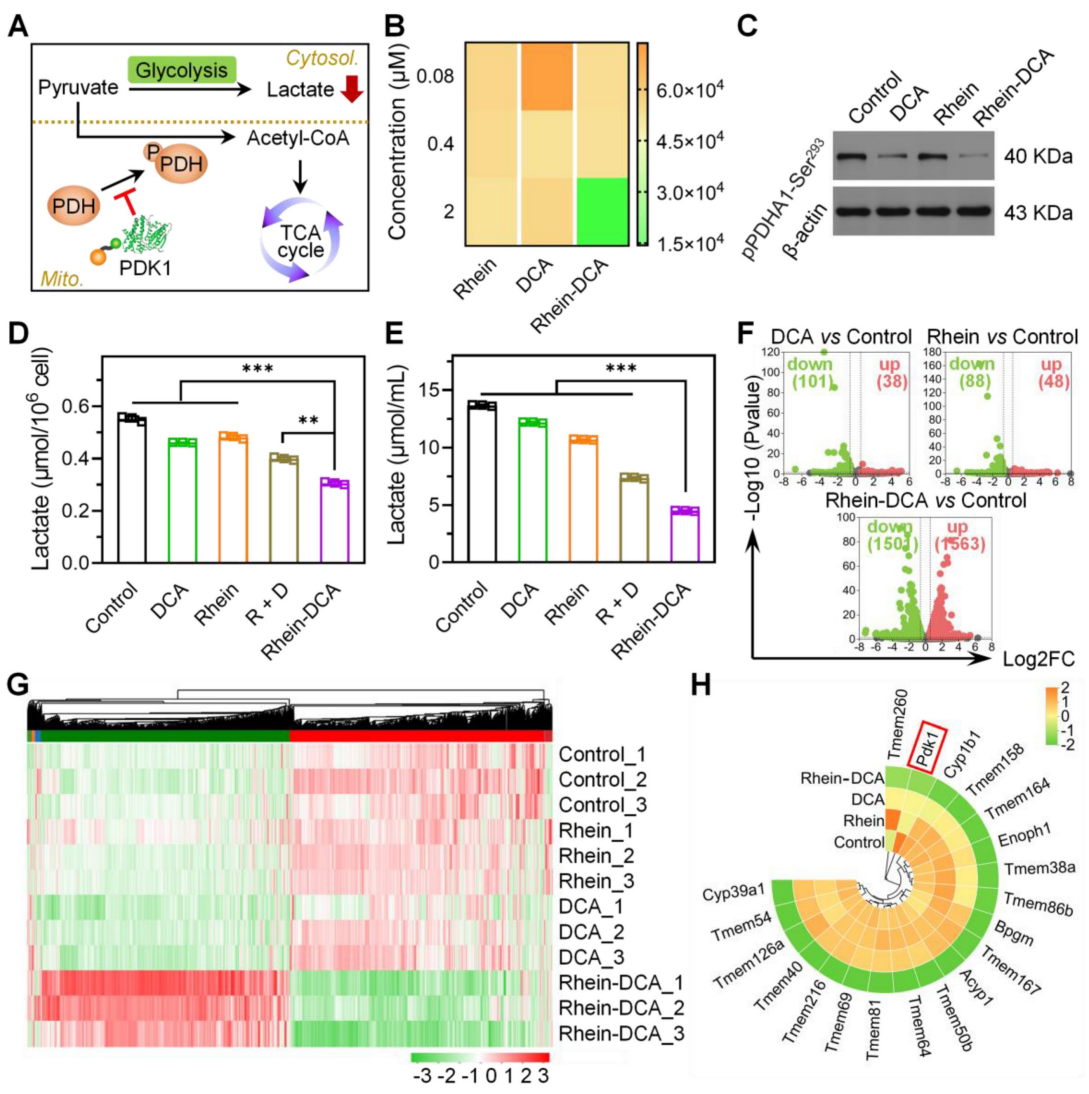
Mitochondrial-targeting Rhein-DCA conjugate inhibited glycolytic metabolic pathway of cancer cells by PDK-PDH axis. A) Illustration of the reversal of the glycolytic phenotype by Rhein-DCA by inhibiting PDK1 activity. B) PDK1 activity assay *in vitro* after various treatments. C) Western bolt analysis of pPDHA1-Ser^293^ protein in 4T1 cells after various treatments. D), E) Intracellular and extracellular lactate levels of 4T1 cells. F) Volcano plots showing the changes of total number of genes when compared between control and DCA-treated, rhein-treated, Rhein-DCA-treated groups, respectively. G) DEGs heatmap analysis. H) Circle heatmap showing the downregulation of glycolysis-related genes. Data represent mean ± S.D., n = 3, p** < 0.01, p*** < 0.001.

The elevated glycolytic metabolism in cancer cells would be blocked when the activity of the gate enzyme PDH was resumed through inhibiting overexpression of PDK1, followed by a decrease in lactate and energy production (Figure 2A). ADP-Glo^TM^ Kinase assay was initially employed to investigate the potential of Rhein-DCA conjugate to suppress PDK1 activity *in vitro*. PDK1 were separately incubated with rhein, DCA, and Rhein-DCA conjugate for 30 min for better binding, then the enzymatic substrate was added to the mixture to initiate the reaction. Finally, the luminescent signal representing PDK1 activity was recorded once the detection procedures were completed. More detailed experimental information was presented in the supporting information. As shown in Figure 2B, it was found that the changes of luminescence in rhein group were almost negligible within the tested concentration range; DCA group also did not exhibit obvious differences; but the luminescent intensity of the solution was dramatically decreased when treated with Rhein-DCA conjugate at 2 μM. The maximum inhibitory effect of Rhein-DCA conjugate on PDK1 suggested that the conjugation of rhein is greatly beneficial for DCA.

Then, the phospho-Ser^293^ PDHA1 protein level in 4T1 cells, which is regulated by PDK1, was further analyzed with western blot (Figure 2C). The results showed that, in contrast to control group, no obvious down-regulated trend was observed in rhein group. A slightly downregulation was appeared in DCA group. Rhein-DCA conjugate group exhibited the lowest expression of phospho-Ser^293^ PDHA1 protein as expected. The results suggested that rhein induced the adequate accumulation of Rhein-DCA conjugate in cellular mitochondria and greatly enhanced the inhibition of PDK1. The conjugate successfully altered glycolysis of 4T1 cells *via* PDK-PDH axis.

Next, the end product of glycolysis, lactate, was analyzed in 4T1 cells to further confirm the efficacy of the Rhein-DCA conjugate in blocking glycolytic metabolism. Intracellular lactate content showed a noticeable descent in Rhein-DCA group, in comparison of other groups with different drug treatments (Figure 2D). Lactate will be released from cells and condense in the TME due to the sustained aerobic glycolysis. To determine whether the decline in intracellular lactate level was caused by an increased extracellular secretion or a disconnect in glycolysis pathway, we further detected the lactate content in the culture medium (Figure 2E). The changes in extracellular lactate level were consistent with those observed intracellularly across the tested groups. These results suggested that the dominant energy supply pathway of glycolysis was totally intercepted by Rhein-DCA conjugate.

To understand the effect of Rhein-DCA conjugate on the energy flow of glycolysis in cancer cells, we performed RNA sequencing analysis on 4T1 cell lines to analyze the genome. After various treatments for 12 h, all samples were clustered *via* principal component analysis (PCA) (Figure S12). The genome cluster heat map of whole genome revealed that Rhein-DCA conjugate treatment disturbed many more genes than DCA or rhein (Figure S13). The statistics of all differentially expressed genes (DEGs) indicated that Rhein-DCA conjugate down-regulated 1501 genes and up-regulated 1563 genes, while rhein only up-regulated 48 genes and down-regulated 88 genes, and DCA up-regulated 38 genes and down-regulated 101 genes (Figure 2F, Figure S14). Furthermore, the DEGs involved in glycolysis pathway of kyoto encyclopedia of genes and genomes (KEGG) databases were analyzed (Figure 2G). We found that glycolysis related genes showed down-regulation of some important genes such as BPGM and ACYP1 in Rhein-DCA group compared to control group. The reported target gene PDK1 of DCA component was down-regulated after DCA treatment. It was surprising that rhein also exhibited down-regulation of the PDK1 gene, which seemed to contradict earlier findings of weak glycolytic effects of rhein, possibly because the ineffectiveness of rhein towards the translated PDK1 protein (Figure 2H). But as a pyruvate analogue, DCA led to the inactivation of PDK1 by binding to the pyruvate-binding pocket of PDK1 protein, together with down-regulation of PDK1 gene, thus leading more prominent restraint of glycolysis. Importantly, in contrast to DCA and rhein, Rhein-DCA conjugate manifested significant down-regulation of PDK1 gene, contributing to genetic disruption of glycolysis.

### Rhein-DCA conjugate inhibited cellular migration

Cellular migration is the pivotal characteristic of cancer metastasis. Researches have suggested that the high level of secreted lactate originating from "Warburg effect" in the TME can promote the metastasis and invasion of tumor cells 29. Considering the inhibition of the glycolysis pathway resulting in a decrease of lactate, we studied the migration behavior of aggressive 4T1 cells *in vitro*. The results of scratch assay were shown in Figure S15, after being treated with different drugs for 24 h, the wound borders of 4T1 cells gradually got closer in control, rhein, DCA and R + D groups, and the migration rates reached approximately 40%. While the migration rate of only 8% in Rhein-DCA group, suggesting that the migratory ability of 4T1 cells was significantly inhibited by Rhein-DCA conjugate. Furthermore, we analyzed the RNAseq data correlate with cellular migration, revealing that Rhein-DCA significantly down-regulated more genetic expression of 4T1 cells, compared with rhein and DCA (Figure S16A). These genes were mainly enriched the cell motility and migration, cell-cell and cell-substrate adhesion terms, exhibiting negative regulation of cell motility (Figure S16B).

### Effects of Rhein-DCA conjugate on OXPHOS inhibition

**Figure 3:**
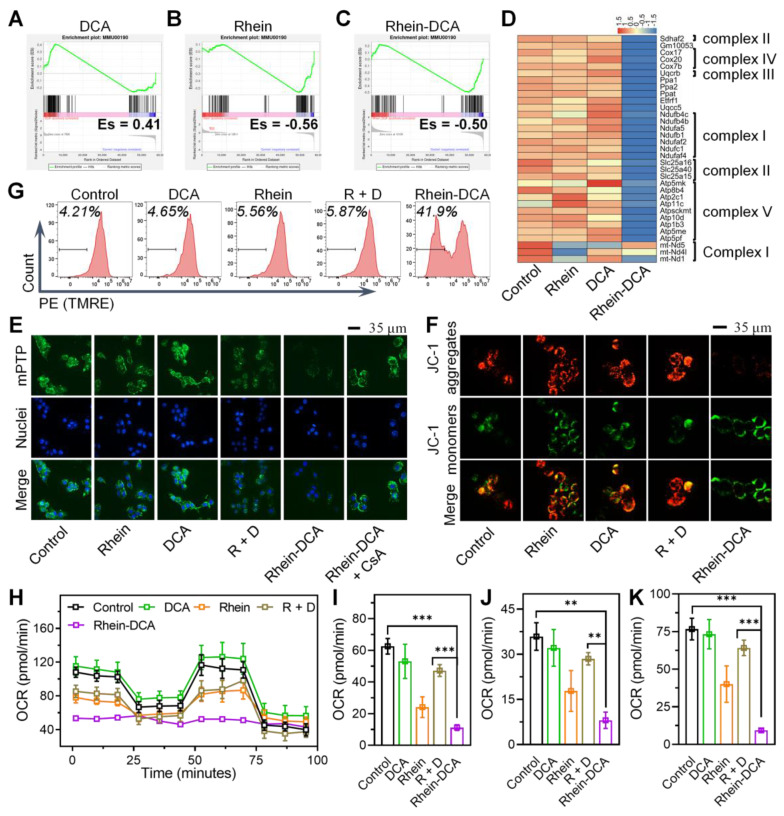
Effects of Rhein-DCA conjugate on mitochondrial OXPHOS metabolism in 4T1 cells. Gene set enrichment analysis (GSEA) plots for OXPHOS pathway between A) DCA *vs* Control, B) Rhein *vs* Control, C) Rhein-DCA *vs* Control. D) Heatmap showing the downregulation of OXPHOS-related genes. E) CLSM images of mPTP in 4T1 cells treated with different drugs. Scale bar represents 35 μm. F) CLSM images of 4T1 cells stained with JC-1 after various treatments. Scale bar represents 35 μm. G) Flow cytometry analysis of 4T1 cells staining with TMRE after different treatments for 24 h. H) Oxygen consumption rate (OCR) of 4T1 cells with various treatments. Calculations of I) basal respiration, J) ATP production, and H) Maximal respiration. Data represent mean ± S.D., n = 3, p** < 0.01, p*** < 0.001.

We next investigated whether the OXPHOS route would be further undermined by Rhein-DCA conjugate when the glycolysis was blocked. The enrichment scores (ES) of greater than zero (ES > 0) in gene set enrichment analysis (GSEA) related to OXPHOS pathway for DCA group *vs* control group, clearly demonstrating that tumor cells could flexibly switch to OXPHOS pathway to continuously obtain ATP for survival, in order to adapt to the energy privation from glycolysis route (Figure 3A). Thus, it was imperative to simultaneously halt glycolysis and OXPHOS to induce energy obstruction and apoptosis. Rhein unit displayed a negative regulation (ES < 0) of OXPHOS signaling pathway (Figure 3B) and down-regulated the mitochondrial genetic expression of mt-ND5, mt-ND4L and mt-MD1 encoding for complex I (Figure 3D), which indicated the feasibility of conjugation of rhein and DCA for lethal energy depletion of cancer cells. As expected, Rhein-DCA conjugate group *vs* control group showed an ES of less than zero (ES < 0), suggesting that cellular OXPHOS energy metabolism was interrupted by Rhein-DCA conjugate (Figure 3C). Gene ontology (GO) term analysis of OXPHOS-associated genes in Rhein-DCA group indicated that downregulated genes mainly focused on the electron transport chain (ETC), NADH dehydrogenase complex assembly, ATP synthase and mitochondrial respiratory chain complex assembly (complex II, III, IV and V) in addition to mitochondrial genome coding for complex I (Figure 3D, Figure S17).

On the other hand, rhein could also disturb mitochondria, the camp of OXPHOS, to further prevent cancer cells from obtaining ATP by inducing pathological opening of mPTP 30. We first detected mPTP on 4T1 cancer cells treated with indicated drugs according to the mPTP assay kit. The green fluorescence of Calcein AM in mitochondria would vanish when the quencher entered mitochondria through the opened mPTP channel. The CLSM images revealed that Rhein-DCA group appeared the faintest green fluorescence among all the tested groups, whereas the fluorescence intensity was restored when the cells were pre-treated with the mPTP inhibitor cyclosporin A (CsA), demonstrating that Rhein-DCA conjugate was valid in inducing the opening of mPTP (Figure 3E). The sustained opening of mPTP along with the loss of Δψm. Thus, we further examined the Δψm of 4T1 cancer cells with JC-1 fluorescence dyes and tetramethylrhodamine, ethyl ester (TMRE) probe. The JC-1 dyes underwent a transition from aggregated red fluorescence to monomeric green fluorescence with mitochondrial depolarization in cells, and the increase of green/red fluorescence intensity ratio of JC-1 dyes indicated mitochondrial depolarization.

DCA did not affect the green and red fluorescence compared to the control group, indicating that mitochondria maintained normal Δψm. Rhein and R + D relatively increased green/red ratio and exhibited relatively decreased Δψm, resulting in mitochondrial damage. But Rhein-DCA conjugate significantly increased the green/red ratio, which was about 7-fold that of control group (Figure 3F, Figure S18A). TMRE is a lipophilic cationic fluorescent probe (Ex = 550 nm, Em = 575 nm). The accumulation of TMRE within the mitochondria would reduce with the mitochondrial depolarization, resulting in a decrease in fluorescence intensity. Flow cytometry results showed that the fluorescence intensity of Rhein-DCA treated 4T1 cells was significantly lower than control, rhein, DCA and R + D groups (*p* < 0.001) (Figure 3G). The percentage of cells that lost TMRE was up to about 40% in Rhein-DCA group, while other groups were about 5% (Figure S18B), further indicating that Rhein-DCA treatment led to significant mitochondrial depolarization. The integrity of mitochondria is significant for mitochondrial bioenergy production. Given the influence of ETC, NADH dehydrogenase, ATP synthase and the decrease of Δψm, we speculated that the ATP production from OXPHOS would be hindered by Rhein-DCA conjugate. Mitochondrial bioenergetic processes of 4T1 cells was measured by oxygen consumption rate (OCR) to reflect the capability of OXPHOS. The overall results shown in Figure 3H, cells treated with Rhein-DCA conjugate exhibited a complete disruption of cellular OXPHOS, including basal respiration, ATP production and maximal respiration (Figure 3I-K). Rhein and R + D appeared moderate effects on the OXPHOS of cells. DCA exhibited the minor effect on OCR profiles. These results demonstrated the alternative OXPHOS energy production pathway of cancer cells could be uncoupled by Rhein-DCA conjugate.

### Dual inhibition of glycolysis and OXPHOS induced energy depletion and apoptosis

**Figure 4:**
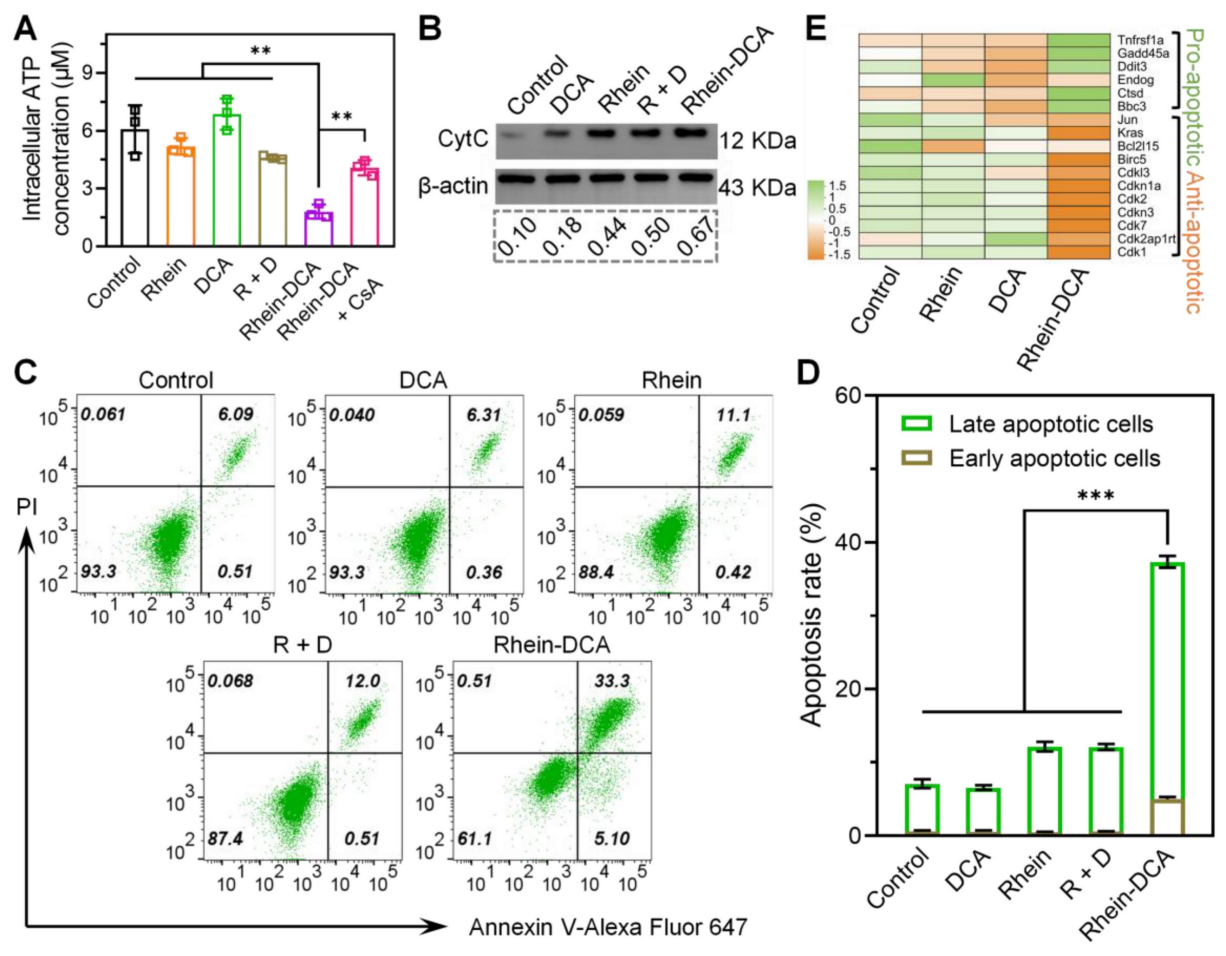
Energy depletion and apoptosis of 4T1 cells induced by Rhein-DCA conjugate. A) Intracellular ATP content in 4T1 cells after various treatments. B) Western bolt analysis of Cyt C protein in 4T1 cells. C), D) Apoptosis of 4T1 cells stained with Annexin V-Alexa Fluor 647/PI. E) Genes expression plots of apoptotic genes from various treatments groups compared to control. Data represent mean ± S.D., n = 3, p** < 0.01, p*** < 0.001.

To demonstrate the effect of the dual-inhibition strategy of Rhein-DCA to cancer cells, the common energetic substance of glycolysis and OXPHOS, the intracellular ATP amount in 4T1 cells was detected. As shown in Figure 4A. Compared with control group, DCA treatment resulted in a slight increase in intracellular ATP amount, which attributed to the reversal of energy metabolism from aerobic glycolysis with poor ATP conversion efficiency to OXPHOS with high ATP synthesis efficiency. Moreover, the addition of rhein caused a decrease in ATP content in cells, suggesting that the ATP generation from OXPHOS was further inhibited. Rhein-DCA conjugate appeared a significant decrease in intracellular ATP content, and CsA treatment could lead to partial recovery of ATP content in cells, indicating that the damage of OXPHOS caused by Rhein-DCA conjugate was partially dependent on the opening of mPTP. Rhein-DCA conjugate achieved synergetic fatal energy depletion in tumor cells. While all compounds exhibited a negligible impact on the energy supply in normal cells (293T) (Figure S19).

The opening of mPTP in mitochondria often leads to the release of pro-apoptotic factor such as cytochrome C (Cyt C), thereby inducing apoptosis 31. Western blot was applied to measure the level of Cyt C in 4T1 cells after treatment with different drugs. The ratio of the integrated density value of target Cyt C protein to the internal parameter β-actin protein was also analyzed (Figure 4B). The released Cyt C in the DCA group could almost be disregarded compared to the control group. The released Cyt C in the rhein and R + D groups was 4.4- and 5.0-fold than that of the control group, respectively, clarifying that rhein component could boost the release of Cyt C in 4T1 cells. The Rhein-DCA group released more Cyt C than rhein and R + D groups. These results indicated that rhein is effective in Cyt C-mediated apoptosis *via* inducing the opening of mPTP, and Rhein-DCA conjugate could further promote the apoptosis.

Correspondingly, the fall of intracellular ATP amount and the release of Cyt C directly drove cells into apoptosis. Flow cytometry apoptosis assay was conducted on the 4T1 cells after culture with different drugs at 6.25 μM for 24 h. The treated cells were stained with Annexin V-Alexa Flour 647 and PI. As shown in Figure 4C-D, there was no obvious changes of apoptosis in DCA group in comparison with control group. Since the antitumor effect could only be achieved when DCA was used in high dose, it is in agreement with reported literature. Similar to rhein group, the apoptosis rate in R + D group increased to approximately 12%. Rhein-DCA group showed the highest cell apoptosis rate, up to 37.4%. Moreover, the genomic data further supported apoptotic results, as shown in Figure 4E, several genes related to apoptosis were differentially expressed. Rhein-DCA conjugate significantly up regulated pro-apoptotic genes involved in p53 signaling pathway, such as BBC3 and GADD45A, and meanwhile down regulated anti-apoptotic genes, such as CDK1, CDK2 and CDKN1A. All results suggested the effectiveness of our dual-starvation therapy strategy.

### Rhein-DCA induced ROS accumulation and triggered ICD* in vitro*

**Figure 5:**
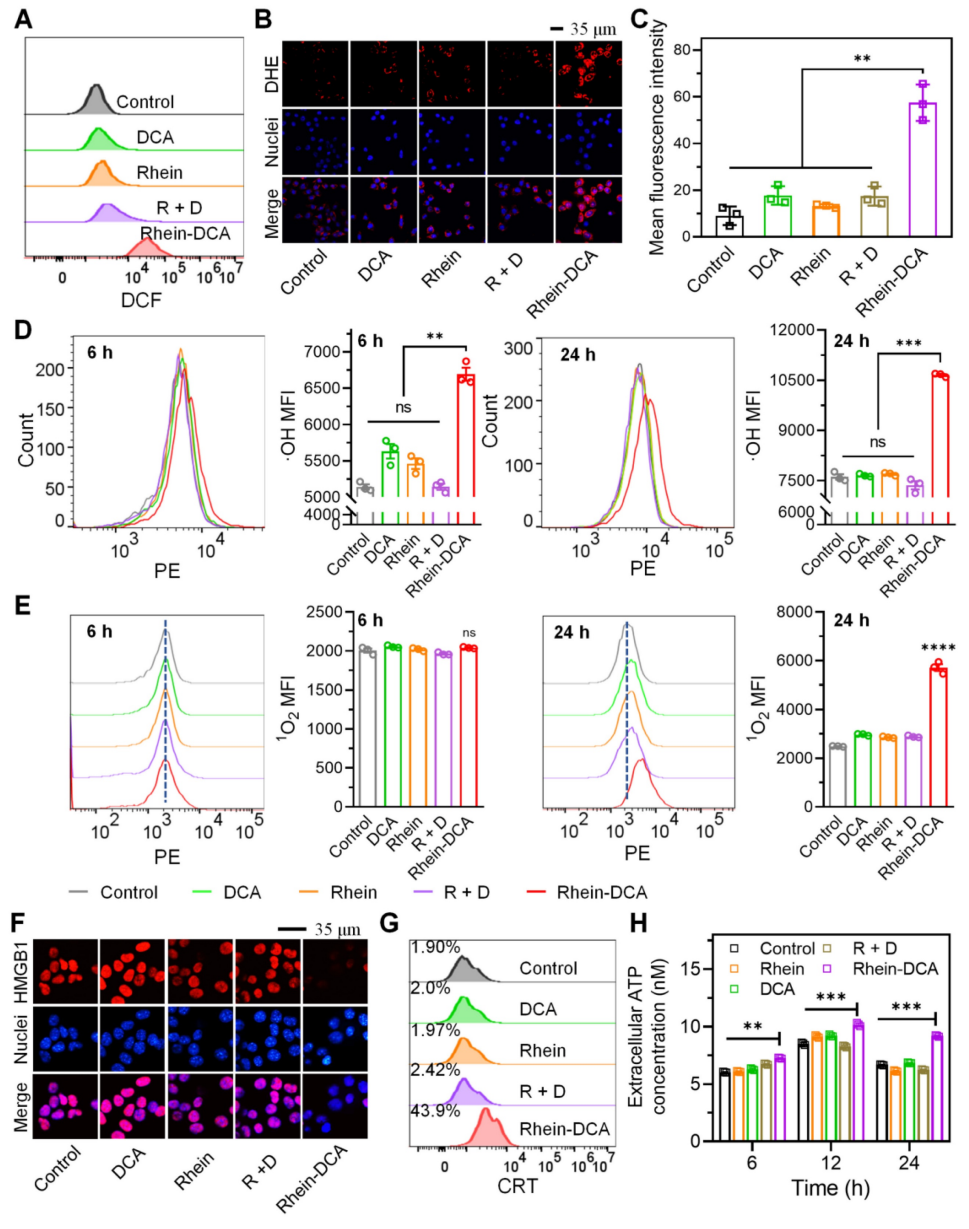
Rhein-DCA conjugate induced ROS accumulation within 4T1 cells and trigger ICD effect *in vitro*. A) Flow cytometry analysis of intracellular ROS production of 4T1 cells staining with DCFH-DA. B) CLSM images and C) the mean fluorescence density analysis of superoxide radio-anionic ROS generation in 4T1 cells staining with DHE. Scale bar represents 35 μm. Flow cytometry analysis and the related mean fluorescence density of intracellular D) ·OH and E) ^1^O_2_ production of 4T1 cells after treated with different treatment for 6 h and 24 h. ICD effect F) CLSM images of HMGB1 protein release from the nucleus of 4T1 cells after different treatments. Scale bar represents 35 μm. G) Flow cytometry analyses of the CRT protein exposure on cells membrane of 4T1 cells. H) Extracellular ATP content in 4T1 cells after different treatments. Data represent mean ± S.D., n = 3, p** < 0.01, p*** < 0.001, p**** < 0.0001.

Rhein is thought to trigger the generation of ROS inside cells by inhibiting mitochondrial complex II, blocking electron transfer from succinate to ubiquinone, which leads to electron leakage at the flavoprotein site 26, 32. Therefore, we investigated the ROS production within 4T1 cells in control, DCA, rhein, R + D and Rhein-DCA groups. Flow cytometry results of 4T1 cells staining with 2',7'-dichlorofluorescein diacetate (DCFH-DA) indicated that the slight enhancement of fluorescence intensity in rhein and R + D groups in contrast to DCA and control groups. It is notable that a stronger fluorescence signal was observed in Rhein-DCA group, which was higher than that in the other groups (Figure 5A), implying that Rhein-DCA conjugate significantly enhanced intracellular ROS level and was more effective than rhein. Then, the cellular superoxide radical anion type of ROS production was further detected using dihydroethidium (DHE) fluorescence dyes after incubation with 4T1 cells. CLSM images displayed that a remarkable red intensity was observed in Rhein-DCA conjugate group among all the tested groups (Figure 5B-C). The flow cytometry was used to further confirm the types of reactive oxygen species, including singlet oxygen (^1^O_2_) and hydroxyl radicals (·OH). The ^1^O_2_ increased greatly about 2.4-fold compared with the control group, and ·OH increased about 1.4-fold (Figure 5D-E). We concluded that the ROS level was greatly increased in Rhein-DCA conjugate group compared with rhein, DCA and R + D groups, which was attributed to assistance of DCA. Due to the conjugating of rhein, the more mitochondrial accumulation and enhanced PDK inhibition activity of DCA promoted the energy metabolism to redirect towards OXPHOS, increased the mitochondrial electron flux, and amplified the generation of ROS. Excessive ROS would cause oxidative damage of mitochondria and further frustrate OXPHOS, leading to the effectively suppress for cancer cells.

It is known that mitochondrial dysfunction occasioned by ROS triggers immunogenic cell death (ICD), characterized by the release of DAMPs from tumor cells, such as high mobility group box 1 (HMGB1), calreticulin (CRT) and ATP. These DAMPs directly reactivated anti-tumor immune response of TAMs of *in vivo* 33. Given the encouraging results of ROS generation, the ICD effect of Rhein-DCA conjugate was consequently explored *in vitro*. HMGB1 is a type of nuclear protein, which would be released to extracellular during the ICD process. CLSM images clearly showed that the red fluorescence of HMGB1 protein was disappeared in Rhein-DCA conjugate group, due to the release of HMGB1 from cell nucleus. While the red fluorescence almost completely located in nuclei in control, rhein, DCA and R + D groups. This was because rhein, DCA, and the R + D cannot induce adequate production of ROS and sufficient ATP depletion (Figure 5F). We further detected the HMGB1 content in the cell culture media with an ELISA kit. The amount of HMGB1 secreted into the culture medium was increased significantly in Rhein-DCA group (*p* < 0.001) compared with control, rhein, DCA and R + D groups. This further confirmed that Rhein-DCA effectively promoted the release of HMGB1 from the cell nucleus to the extracellular space (Figure S20). Unlike HMGB1, abundant CRT often exposed on the cell membrane surface of tumor cells. Flow cytometry was employed to analyze CRT protein of 4T1 cells. Cells treated with Rhein-DCA exhibited significant increase of surface-exposed CRT (44%) compared to other groups (Figure 5G). The secreted ATP from 4T1 cells with immunogenicity was further measured to evaluate the ICD effect after being cultured with various drugs for 6, 12 and 24 h. As shown in Figure 5H, the level of extracellular ATP in supernatants of 4T1 cells was higher after Rhein-DCA treatment compared to the control, rhein, DCA and R + D groups from 6 h to 24 h. There was no apparent distinction between rhein, DCA, R + D groups and the control group when co-cultured for 6 or 12 h. A slight difference was observed until the cultivation time was prolonged to 24 h. Thus, the results suggested that Rhein-DCA conjugate was a novel ICD-inducer based on mitochondrial-targeted ROS stress, responsible for the release of immunogenic DAMPs. This reminded us that the Rhein-DCA conjugate may enhance tumor treatment by promoting ICD-mediated immunotherapy.

### Antitumor effects and biosafety *in vivo*

The proven oral administration experiences of rhein and DCA validated the feasibility and potential of our research on the oral administration of their derivative, Rhein-DCA 34, 35. We first evaluated the stability of Rhein-DCA by simulating gastric acid (pH 1.5) and intestinal (pH 6.8) conditions *in vitro* with HPLC. The stability of the Rhein-DCA was as high as 85% within 2 h, indicating the stable of Rhein-DCA in gastrointestinal environment, and it was capable of being efficiently absorbed *via* the gastrointestinal tract (Figure S21).

**Figure 6:**
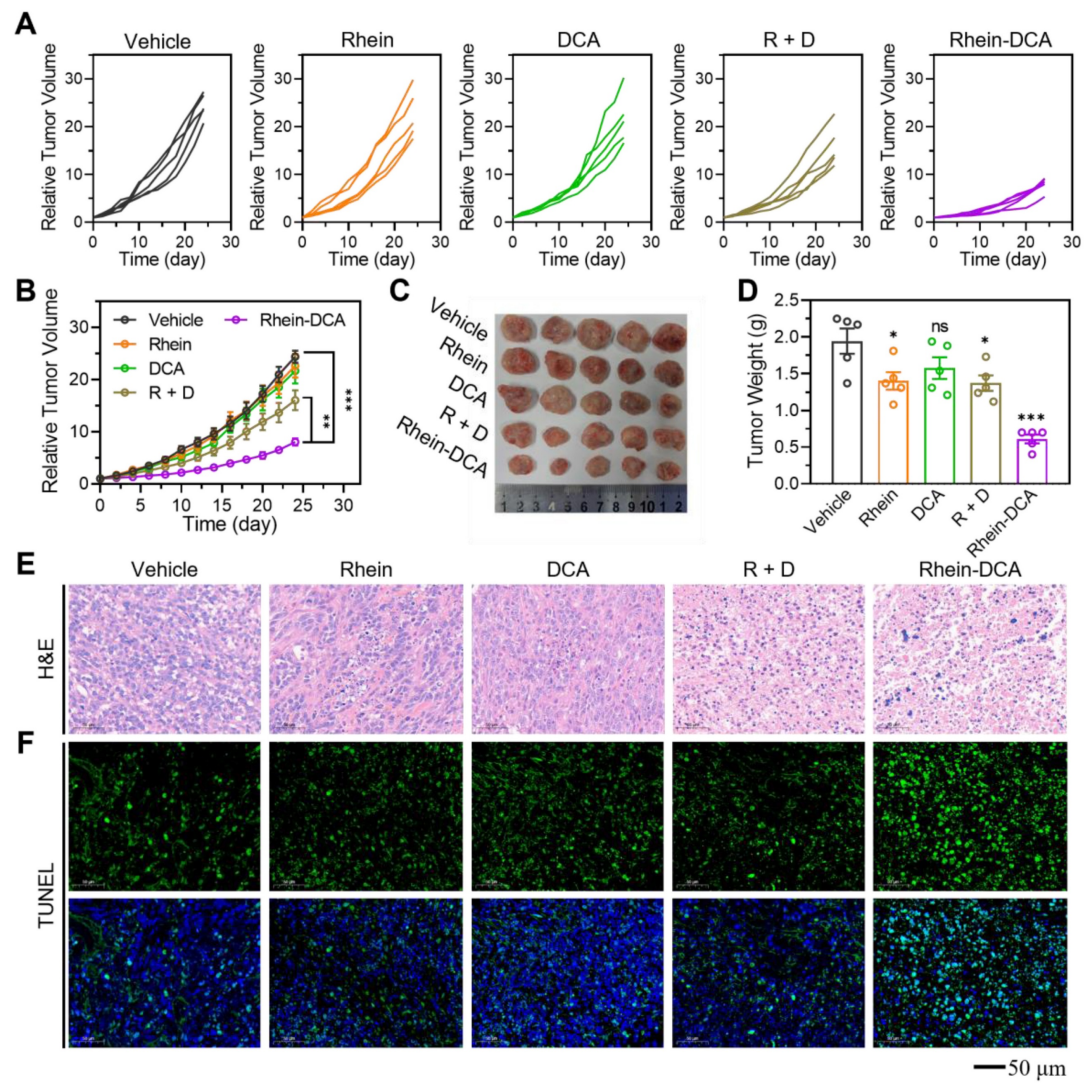
*In vivo* antitumor effect on 4T1 xenograft tumor model. A) The individual tumor volume change profiles of each mouse in the vehicle, rhein, DCA, R + D and Rhein-DCA groups. B) The tumor average grown profiles of 5 mice in various groups, respectively (n = 5). C) D) Photographs and related weights of excised tumors of different groups (n = 5). E) F) H&E staining and TUNEL staining of tumor tissue after vehicle, rhein, DCA, R + D and Rhein-DCA conjugate treatments. Scale bar represents 50 μm. Data represent mean ± S.D., n = 3, p* < 0.05, p** < 0.01 p*** < 0.001.

The antitumor efficiency of Rhein-DCA conjugate *in vivo* was explored using the female Balb/c mice with 4T1 tumor xenografts model. Briefly, 4T1-tumor bearing mice were randomly divided into vehicle, rhein, DCA, R + D and Rhein-DCA conjugate groups when the tumor became apparent (n = 5). Mice were treated daily with vehicle (2.85% anhydrous ethanol / 10% cremophor / 0.02% 0.1 M HCl / 87.15% saline) and rhein, DCA, R + D and Rhein-DCA conjugate (20 mg/kg) by oral gavage for 24 days. The body weight and tumor volume of mice were monitored and recorded every two days. The tumor growth curves of 4T1 tumors suggested that rhein and DCA induced a slight inhibition in tumor growth compared with the vehicle group. The combined treatment group (R + D) exhibited a moderate reduction in tumor growth. Notably, the Rhein-DCA conjugate demonstrated a significant inhibition in tumor growth, and the tumor size significantly decreased compared with the vehicle group (*p* < 0.001) after 24 days of treatment (Figure 6A-B). The photograph and the weight information of all excised tumor xenografts further visually confirmed the antitumor effect of Rhein-DCA conjugate *in vivo* (Figure 6C-D). Next, the hematoxylin-eosin (H&E) assay and TdT-mediated dUTP Nick-End Labeling (TUNEL) staining further revealed only mild apoptosis and necrosis in rhein, DCA and R + D groups, likely due to insufficient targeting of DCA, and limited bioavailability of rhein. As expected, Rhein-DCA conjugate led to more extensive tissue necrosis and considerable cell apoptosis than the other groups (Figure 6E-F). Due to suppression of tumor growth, the survival of mice treated with Rhein-DCA conjugate was improved in contrast to vehicle group (Figure S22). These results demonstrated the positive antitumor effects of Rhein-DCA conjugate by off energizing cancer cells.

Next, we evaluated the biosafety of Rhein-DCA conjugate *in vivo*. The weight curves indicated that no prominent changes of mice body weight were appeared in any treatment, indicating that compounds showed minimal toxicity to the mice (Figure S23). The blood biochemistry indexes, including alanine aminotransferase (ALT), aspartate aminotransferase (AST), creatinine (CREA) and urea (UREA) were detected after 24-days treatment to confirm the functions of liver and renal (Figure S24). The values of above indicators remained within normal ranges in all groups. These findings supported the biosafety of the Rhein-DCA conjugate and its components, even at therapeutic doses.

### Lactate decline and ICD effect induced immune response of macrophages *in vivo*

**Figure 7:**
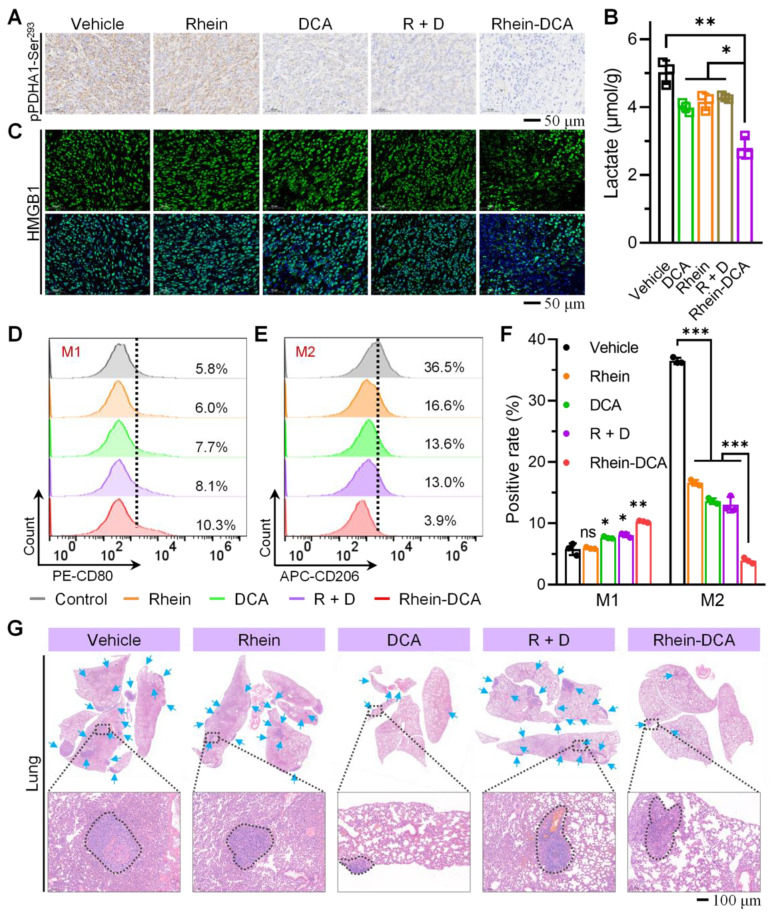
*In vivo* antitumor effect of Rhein-DCA on 4T1 xenograft tumor model. A) IHC staining of pPDHA1-Ser^293^ protein in tumor slices after different treatments. Scale bar represents 50 μm. B) The lactate content in tumor tissues from mice treated as indicated compounds measured with an ELISA kit. C) IF staining of HMGB1 protein on 4T1 tumor slices after different treatments. Scale bar represents 50 μm. Flow cytometry plots CD11b-positive and D) CD80-positive M1-phenotype macrophages and E) CD206-positive M2-phenotype macrophages in the primary tumors after various treatments. F) Quantification of the M1 (CD80^+^ in CD11b^+^ cells) and M2 macrophage positive rate (CD206^+^ in CD11b^+^ cells) according to the flow cytometric results. G) H&E staining of whole lungs after vehicle, rhein, DCA, R + D and Rhein-DCA conjugate treatments. Scale bar represents 100 μm. Data represent mean ± S.D., n = 3, p* < 0.05, p*** < 0.001.

The TAMs are the prominent components in the TEM and play a critical role in tumoral immunosuppression. Repolarization of protumor M2 phenotype into antitumor M1 phenotype is promising for malignant tumors 36. On the one hand, as a key metabolite fueling M2 polarization, lactate is very important in modulating the polarization state of macrophages within the tumor microenvironment 20. To verify that Rhein-DCA conjugate reprogrammed the metabolism of cells on solid tumors, we analyzed the expression of pPDHA1-Ser^293^ protein in tumor tissues with immunohistochemical staining (IHC) (Figure 7A). The pPDHA1-Ser^293^ protein level showed obvious down-expression in DCA group compared to the vehicle group, thus confirming its established role in regulating PDK-PDH axis to inhibit glycolysis. The end product lactate of glycolysis decreased 20%, consistent with DCA's ability to reverse the Warburg effect. While rhein did not significantly alter the level of pPDHA1-Ser^293^, interestingly, it reduced tumor lactate levels, which may be attributed to an inhibitory effect on glycolysis through HIF-1α-mediated pathways, as previously reported 37. The R + D group showed similar effects to DCA monotherapy, indicating no synergistic interaction between the two free drugs. Notably, the Rhein-DCA conjugate achieved a significant dual metabolic disruption: the pPDHA1-Ser^293^ protein level showed down-expression compared with vehicle group, and lactate levels plummeted to 44% of the vehicle group (*p* < 0.01), suggesting that mitochondria-targeting Rhein-DCA conjugate achieved an effective block of glycolytic energy metabolism and contributed to the energy depletion and lactate decrease *in vivo* (Figure 7B). On the other hand, the ICD behavior of cancer cells also can activate TAMs into M1 polarization. Then, the release of HMGB1 was evaluated to assess the ICD effect *in vivo* with immunofluorescence staining (IF) (Figure 7C). The green fluorescence of HMGB1 mostly appeared outside the nucleus with weak intensity in Rhein-DCA group, indicating active release of this danger-associated molecular patterns (DAMPs) into the extracellular space. In contrast, almost all of HMGB1 overlapped with nucleus in vehicle, rhein, DCA and R + D groups, suggesting minimal ICD induction. The decrease HMGB1 in primary tumor demonstrated that mitochondrial disturbing-evoked ICD effect by Rhein-DCA conjugate *in vivo*, and highlighted the necessity of Rhein-DCA conjugate to achieve subsequent immune activation.

To further investigate the immunomodulatory effects of the Rhein-DCA conjugate, we analyzed TAMs by flow cytometry, where CD11b and CD80 antibodies were labeled for M1-phenotype macrophages (Figure 7D), CD11b and CD206 antibodies were labeled for M2-phenotype macrophages (Figure 7E). In comparison of vehicle group (M1: 5.8%, M2: 36.5%), rhein selectively reduced M2 macrophages to 16.6% (*p* < 0.001), but failed to activate M1 polarization (6.0%, *p* > 0.05). DCA modestly increased M1 macrophages (7.7%, *p* < 0.05) and reduced M2 macrophages (13.6%, *p* < 0.001), attributable to its ability to deplete lactate, a key metabolite fueling M2 polarization. R + D group exhibited limited synergy, suggesting the inefficacy of single drug coadministration. It was satisfactory that the Rhein-DCA conjugate achieved a dual immunomodulatory effect: M1 macrophages increased to 10.3% (1.8-fold *vs* vehicle, *p* < 0.001), while M2 macrophages decreased to 3.9% (9.4-fold reduction *vs* vehicle, *p* < 0.001) (Figure 7F). These results indicated Rhein-DCA conjugate treatment achieved the potent immune response *in vivo* by inducing M1 polarization of TMAs and eliminating M2 macrophages in primary tumors based on mitochondrial damage causing-ICD and reduction of secreted lactate.

Correspondingly, the lung and liver tissues, which are the most common sites for primary tumor metastasis, were obtained and imaged, and then subjected to H&E staining for histopathological examination. The digital images of whole lungs and livers revealed that visible metastatic nodule of large size was clearly observed in vehicle, rhein, DCA and R + D groups, whereas Rhein-DCA conjugate significantly decreased the number and size of metastatic lesions (Figure S25). H&E images provided more detailed information, lesions were greatly decreased in the lung of Rhein-DCA conjugate group in contrast to the other groups (Figure 7G, Figure S26), demonstrating that macrophages repolarization successfully enhanced immune response *in vivo* and eventually inhibited primary tumor growth and metastasis.

### Pharmacokinetic study and tissue distribution

HPLC was conducted to evaluate the absorption and distribution of Rhein-DCA conjugate in mice after oral administration. Rhein-DCA was orally administered to Sprague-Dawley (SD) rats at 20 mg/kg, and the concentration of the drug in the blood samples was serially measured at predefined time points. The plasma concentration AUC(0-∞) was calculated as 8.98 μg h/mL, and a favorable maximum concentration (C_max_ = 2.68 μg/mL). Rhein-DCA exhibited a good plasma clearance (CL = 2.25 L/h/kg) and an elimination half-life (t_1/2_) of 5.54 h in oral gavage administration (Table S1).

4T1-bearing Balb/c mice were orally administered with Rhein-DCA (20 mg/kg), major organs (heart, liver, spleen, lung, kidney) and tumor tissues were harvested at 2, 6, and 24 h after administration. Rhein-DCA conjugate was detectable in tumors at 2 h after treatment, with concentrations peaking at 6 h and remaining quantifiable up to 24 h, indicating a prolonged intra-tumoral retention. The Rhein-DCA conjugate could be absorbed rapidly and broadly distributed in major organs tissue after administration. By 24 h, systemic clearance from various major organs was extremely completed (Figure S27).

## Conclusion

In summary, we developed a novel mitochondria-targeting small-molecule inhibitor consisting of glycolysis inhibitor DCA and OXPHOS inhibitor rhein to off energize cancer cells and induced macrophage repolarization *in vivo* for efficient synergetic cancer treatment. Due to the introduction of rhein fragment, the conjugate Rhein-DCA efficiently accumulated in mitochondria and enhanced the inhibition of PDK activity, leading to a complete disaster of the entire glycolytic pathway by abolishing PDK-PDH axis *in vitro* and *in vivo*. RNAseq analysis demonstrated that the conjugation of DCA and rhein enabled Rhein-DCA conjugate impeded alternative OXPHOS metabolic pathway. The continuous opening of mPTP induced by rhein unit in Rhein-DCA conjugate decreased Δψm, which further prevented cancer cells from obtaining ATP from OXPHOS. Rhein-DCA conjugate simultaneously cut off ATP supplement of cancer cells from OXPHOS and glycolysis and induced apoptosis. In addition, Rhein-DCA conjugate-induced ROS elevation could trigger the release of DAMPs, together with low lactate, eventually activated anti-tumor immune responses of TAMs. After oral administration, the Rhein-DCA exhibited significant therapeutic effect and impeded tumor metastasis *in vivo*. This work provided a single small-molecule to co-destroy double energy metabolism pathways of OXPHOS and glycolysis for cancer therapy, offering new insights for studies of small molecule drugs in metabolic therapy.

## Methods

### Synthesis of Rhein-EG

Rhein (0.5 g) was added to the EG (50 mL) and the reaction mixture was magnetically stirred and heated to 95 ^o^C. H_2_SO_4_ (2 mL) was added dropwise and the mixture was stirred at 95 °C for 6 h. Dichloromethane (CH_2_Cl_2_) was added to the mixture to extract a yellow liquid, which was washed several times with H_2_O, saturated NaHCO_3_ and H_2_O in sequence, and dried with anhydrous sodium sulphate. The solvent of dichloromethane was removed by evaporation under reduced pressure and a yellow solid (Rhein-EG) was obtained. The structure and purity were confirmed with ^1^H NMR, (400 MHz, DMSO-*d*_6_, ppm): δ = 11.82 (s, 2H), 8.04 (s, 1H), 7.81-7.77 (m, 2H), 7.67 (d, J = 7.6 Hz, 1H), 7.38 (d, J = 8.4 Hz, 1H), 5.05 (s, 1H), 4.34 (t, J = 4.8 Hz, 2H), 3.77-3.74 (m, 2H). ^13^C NMR (101 MHz, DMSO-*d*_6_, ppm): δ = 191.57, 181.14, 164.39, 161.88, 161.44, 138.11, 137.11, 134.16, 133.45, 125.12, 124.67, 119.92, 119.24, 119.00, 116.45, 68.06, 59.36. HRMS (ESI): *m/z* Calcd for C_17_H_11_O_7_^-^: 327.0510; found: 327.0509.

### Synthesis of Rhein-DCA

To a suspension of Rhein-EG (0.15 g) in anhydrous CH_2_Cl_2_ (220 mL), triethylamine (0.125 mL) and dichloroacetyl chloride (0.0438 mL) were added, and the reaction mixture was stirred under nitrogen atmosphere at 0 °C for 24 h. Then, the mixture was washed several times by H_2_O, 0.5 M HCl, saturated NaHCO_3_ and H_2_O in turn. The solvent was removed by evaporation under reduced pressure. The solid obtained was re-dissolved in CH_2_Cl_2_ and purified by column chromatography on silica gel (300 mesh) eluted with CH_2_Cl_2_/CH_3_OH (500:1, v/v). The structure and purity were confirmed with ^1^H NMR (400 MHz, DMSO-*d*_6_, ppm) δ = 11.86 (s, 2H), 8.08 (d, J = 1.6 Hz, 1H), 7.84-7.80 (t, 1H), 7.74-7.69 (m, 2H), 7.39 (dd, J = 8.4, 1.2 Hz, 1H), 6.96 (s, 1H), 4.68-4.61 (m, 4H). ^13^C NMR (101 MHz, DMSO-*d*_6_, ppm): δ = 191.63, 181.20, 165.05, 164.12, 161.88, 161.41, 138.12, 136.57, 134.37, 133.55, 125.13, 124.57, 119.94, 119.59, 118.97, 116.59, 65.34, 65.32, 63.69. HRMS (ESI): *m/z* Calcd for C_19_H_11_Cl_2_O_8_^-^: 436.9836; found: 436.9829.

### Stability of Rhein-DCA

UV-Vis spectra were used to investigate the stability of Rhein-DCA conjugate. The Rhein-DCA was dispersed in 2.5% DMSO-PBS at room temperature, and the absorption spectrum was monitored at 0, 2, 6 and 12 h. High performance liquid chromatography (HPLC) was used to investigate the intracellular stability of Rhein-DCA conjugate. 4T1 cells were seeded in cell culture dish (diameter of 10 cm) at a density of 2 × 10^6^ cells per dish and cultured in the incubator for 24 h. Then the cells were further treated with Rhein-DCA conjugate at a concentration of 11 μg/mL for 4 h. After removing media and washing the cells three times with PBS to remove the residual Rhein-DCA, the cells were incubated for another 2 h. Then, the cells were collected and washed twice with PBS. The cells were suspended in 1 mL methanol to obtain cell lysates by three freeze-thaw. Then the supernatant was collected after centrifugation at 13000 G for 10 min for HPLC analysis. A C18 column was used with methanol / 0.1% H₃PO₄ (83:17) as the mobile phase, flow rate 1.0 mL/min, fluorescence detector with an excitation wavelength of 443 nm and an emission wavelength of 530 nm.

### Cell lines and culture conditions

Mouse breast cancer 4T1 cells and Mouse colorectal carcinoma CT26 cells were purchased from Haixing Biosciences Co., Ltd (Jiangsu, P. R. China), and cultured in complete RPMI-1640 medium supplemented with 10% FBS and 1% Penicillin / Streptomycin in a cell incubator (37 °C in 95% air and 5% CO_2_ atmosphere). Human breast cancer MCF-7 cells, Human cervical carcinoma HeLa cells and Human embryonic kidney 293T cells were obtained from the China Center for Type Culture Collection (CCTCC). MCF-7 and HeLa cells were cultured in DMEM medium containing 10% FBS and 1% Penicillin / Streptomycin and then cultured in a cell incubator (37 °C in 95% air and 5% CO_2_ atmosphere).

### Cell uptake and localization assay

4T1 cells were seeded in confocal dishes at a density of 10 × 10^4^ cells per dish and cultured in the incubator for 24 h. And the cells were further treated with Rhein-DCA conjugate at a concentration of 6.25 μM for 0, 2, 6, 12 or 24 h, respectively. Thereafter, the cells were washed twice with PBS and stained with Hoechst 33342 (10 μg/mL) for 15 min in the dark at room temperature, followed by another two washes with PBS. Finally, the fluorescence intensity of cells was monitored with confocal microscope with excitation wavelengths (Ex) of 488 nm, emission wavelengths (Em) of 525 ± 25 nm, and Ex of 405 nm, Em of 447 ± 30 nm, respectively.

To investigate the mitochondrial accumulation of Rhein-DCA conjugate in cancer cells, after being incubated with Rhein-DCA conjugate for 24 h, 4T1 cells were stained by Mito Tracker Deep Red FM (Ex = 644 nm, Em = 665 nm), Lyso Tracker Red (Ex = 577 nm, Em = 590 nm) and ER Tracker Red (Ex = 587 nm, Em = 615 nm) for 30 min at 37 °C and 5% CO_2_. Then, cells were washed twice with PBS for imaging by confocal microscope.

### Cell cytotoxicity *in vitro*

The cytotoxicity of rhein, DCA, the mixture of rhein and DCA (R + D) and Rhein-DCA conjugate was evaluated on HeLa, MCF-7, CT26, 4T1 and 293T cell lines by MTT assay. Cells were seeded in 96-well plates at a density of 3 × 10^3^ cells per well in the corresponding complete culture medium and cultured for 24 h. Then cells were incubated with 200 μL fresh medium containing the respective desired concentrations of rhein, DCA, R + D (with the concentrations being 100, 66.7, 44.4, 29.6, 19.8, 13.2, 8.8, 5.9, 3.9, 2.6, 1.7 and 1.2 μM, respectively) and Rhein-DCA conjugate (with the concentrations being 29.6, 19.8, 13.2, 8.8, 5.9, 3.9, 2.6, 1.7, 1.2, 0.8, 0.5 and 0.3 μM, respectively). After 72 h of incubation, 20 μL of MTT solution (5 mg/mL) was added to each well and the plates were cultured for another 4 h. The medium was carefully removed, and 110 μL of DMSO was added into per well to dissolve the formazan. The absorption densities at 590 nm were recorded with microplate reader. The quoted IC_50_ values were presented as the mean ± standard deviation.

### PDK1 kinase activity assay

The inhibitory effect of PDK1 kinase was evaluated with the ADT-Glo kinase assay. Firstly, PDK1 enzyme, PDK tide, ATP, and indicated drugs (dissolved in DMSO) were dissolved in kinase buffer. Then, 5 μL of compounds (rhein, DCA and Rhein-DCA) with diverse concentrations and 10 μL of PDK1 enzyme were dispensed to the 96-well plate and incubated for 30 min at room temperature. Next, 10 μL of PDK tide/ATP mixture was added and incubated at room temperature for 60 min. Finally, to halt the reaction and deplete the remaining ATP, 25 μL of ADP-Glo™ reagent was added to each well and incubated at room temperature for 40 min. 50 μL of kinase detection reagent was added and incubated at room temperature for another 30 min. Ultimately, the luminescence signal was recorded by multi-mode microplate reader (Integration time: 1 s). The concentrations of reagents employed in this assay were as follows:

### Western blot assay

4T1 cells were seeded in 6-well plates at a density of 20 × 10^4^ cells per well and cultured for 24 h. Then the cells were treated with rhein, DCA, R + D and Rhein-DCA conjugate, each at a concentration of 6.25 μM for 24 h. Cells were washed twice with PBS and lysed by RIPA total protein extraction solution containing multiple protease inhibitors for 30 min while being maintained on ice. The lysate was centrifuged at 9391 G for 10 min at 4 °C to obtain the total protein solution. The concentrations of total protein were determined with BCA Protein Assay Kit. The protein was further denatured with 5 × protein loading buffer at 100 °C for 15 min. Sodium dodecyl sulfate polyacrylamide gel electrophoresis was conducted to separate the protein and transferred it to PVDF membranes. The PVDF membranes were blocked with 5% skim milk for 30 min at room temperature and incubated with indicated primary antibodies: Anti-Cytochrome C Rabbit pAb (1:1000); Anti-β-actin Rabbit pAb (1:1000); or Anti-PDHA1 Rabbit mAb (phosphoS^293^) (1:1000) overnight at 4 ^o^C. The membranes were incubated with HRP conjugated Goat Anti-Rabbit IgG (H+L) (1:5000), respectively, for 30 min at room temperature and then analyzed by chemiluminescence (Clinx,6100).

### Intracellular and extracellular lactate determination

4T1 cells were seeded into 6-well plates at a density of 10 × 10^4^ cells per well and cultured for 24 h. Cells were exposed to PBS, rhein, DCA, R + D and Rhein-DCA conjugate at a concentration of 6.25 μM for 24 h. (1) Medium: The medium from different groups was subjected to centrifugation at 9391 G for 10 min, 100 μL of supernatant was obtained, and 1 mL of extraction solution I was added. (2) Cells: After being washed twice with PBS, cells were harvested and suspended in extraction solution I (1 mL) for further lysis with sonication in ice bath (power 300 W, the duration of sonication is 3 s, the interval duration of 7 s, total sonication time of 3 min). The extraction solutions obtained from both the medium and cells lysate were centrifuged at 12000 G for 10 min at 4 ^o^C, yielding 0.8 mL of supernatant. Next, 0.15 mL of extraction solution II was slowly added and mixed until no bubbles were present. The mixture was centrifuged at 12000 G for 10 min at 4 ^o^C, and the supernatant was collected for analysis. Subsequently, 40 μL of reagent I, 10 μL of reagent II and 20 μL of reagent IV were added to 10 μL of above supernatant and incubated at 37 °C for 20 min. After that, 6 μL of reagent V and 60 μL of reagent III were added and the incubation was continued at 37 °C for 20 min in the dark. The resulting mixture was centrifuged at 12000 G for 10 min at 25 °C to obtain the precipitate, which was then dissolved in 200 μL of ethanol. Finally, the absorption value of each sample was detected with microplate reader at 570 nm.

### RNA sequencing and analysis

4T1 cells were seeded in 10 cm culture dishes and cultured until the confluency reached approximately 80%. The cells were treated with PBS, DCA, rhein, and Rhein-DCA conjugate (at a concentration of 6.25 μM) for 12 h. After that, the cells were collected and suspended in TRIzol Reagent for RNA sequencing. All samples were sent to Shanghai Majorbio Bio-pharm Biotechnology Co., Ltd. (Shanghai, P. R. China) for detection. Total RNA was extracted from 4T1 cells and was measured using the nanodrop-2000. The quality of RNA was evaluated by agarose gel electrophoresis and analyzed with Agilent5300. cDNA libraries were constructed according to the Illumina stranded mRNA prep, Ligation (USA) protocol. The DNA was further enriched by PCR amplification and purified by 2% Low Range Ultra Agarose. Clusters were generated by Bridge PCR on the cBot after being quantified by Qubit 4.0, and sequenced with NovaSeq X Plus platform (PE150). The differential expression analysis of various gene expressions was performed using DESeq2. The GO functional enrichment and KEGG pathway analysis were conducted on Goatools and Python scipy software.

### Scratch assay

4T1 cells were seeded in 6-well plate and incubated for 24 h. A straight scratch line across the cell monolayer was marked at the bottom of the plate with 200 μL pipette tip. Plate was washed twice with PBS to remove the detached cells. 6.25 μM of indicated drugs were added into each well, then the wound borders of cells were imaged at 0 h, 12 h and 24 h, respectively. The migration rate was calculated as follows: migration rate (%) = (initial scratch area - final scratch area) / initial scratch area × 100%, and the scratch area was processed with the Image J derivatives Fiji software.

### Mitochondrial permeability transition pore opening assay

The opening status of the mPTP in 4T1 cells were tested with fluorescent probe Calcein AM. Briefly, 4T1 cells were seeded into 6-wells plates at a density of 10 × 10^4^ cells per well and treated with various drugs at 6.25 μM for 24 h. After being washed twice with PBS, the cells were stained with Calcein AM staining solution (1 ×) and CoCl_2_ fluorescence quenching solution (1 ×) for 45 min at 37 °C in the dark. Next, they were further cultured with fresh medium at 37 °C for another 30 min in the dark. The cellular nuclei were stained with Hoechst 33342 for 10 min at room temperature. Finally, the images of cells were captured with confocal microscope after washed twice with PBS again.

### Mitochondrial membrane potential assay

JC-1: The Δψm was performed using the JC-1 assay with flow cytometry and confocal microscope. 4T1 cells were separately seeded into 6-well plates and confocal dishes at a density of 10 × 10^4^ cells per well and cultured at 37 °C for 24 h. Cells were cultured with PBS, rhein (6.25 μM), DCA (6.25 μM), R + D (6.25 μM + 6.25 μM) and Rhein-DCA (6.25 μM) for another 24 h. Following staining with JC-1 dye (1 ×) for 20 min at 37 °C according to the protocol, the cells and washed twice with precooled JC-1 staining buffer (1 ×) for detection. Samples were then immediately detected by flow cytometry and imaged with confocal microscope.

TMRE: 4T1 cells were separately seeded into 6-well plates at a density of 10 × 10^4^ cells per well and cultured at 37 °C for 24 h. Cells were cultured with PBS, rhein (6.25 μM), DCA (6.25 μM), R + D (6.25 μM + 6.25 μM) and Rhein-DCA (6.25 μM) for another 24 h. Following staining with TMRE dye (1 ×) for 15 min at 37 ^o^C, the cells were washed twice with complete RPMI-1640 medium for detection. Samples were then immediately detected by flow cytometry.

### Oxygen consumption rate

The mitochondrial bioenergetics of 4T1 cells was investigated by Seahorse Extracellular Flux analyser. 4T1 cells were seeded in 24-well plates (Seahorse Bioscience) at a density of 2 × 10^4^ cells per well and incubated for 24 h at 37 °C in 5% CO_2_ atmosphere. The cells were treated with indicated drugs at a concentration of 6.25 μM for 24 h. At the same time, XF sensor cartridges were hydrated overnight in a CO_2_-free environment. The cells were cultured in the incubator for 1 h at 37 °C without CO_2_ after washed with Seahorse XF assay medium. Three baseline measurements of OCR were detected with Seahorse XFe24 analyzer when the oligomycin (1.0 μM, ATP synthase complex inhibitor), FCCP (1.0 μM, ATP synthesis uncoupler trifluorocarbonylcyanide phenylhydrazone) and the mixture of antimycin-A (1.0 μM, inhibitor of complex III) and rotenone (1.0 μM, inhibitor that prevents the transfer of electrons from the Fe-S center in complex I to ubiquinone) were sequentially injected for the detection of the oxygen consumption rate.

### Intracellular ATP detection

The intracellular ATP content was measured with PhosphoWorks™ Colorimetric ATP Assay Kit. Specifically, 4T1 cells and 293T cells were seeded in 12-well plates at a density of 2 × 10^5^ cells per well and further cultured for 24 h at 37 °C in an atmosphere of 95% air and 5% CO_2_. For the control group, the culture medium was replaced with fresh medium. For the rhein, DCA, R + D, and Rhein-DCA groups, the medium was replaced with fresh medium containing the indicated drugs at a concentration of 6.25 μM. It should be noted that in the Rhein-DCA + CsA group, the cells were pretreated with CsA (2.5 μM) for 1 h at 37 °C before being cultured with Rhein-DCA. The cells were collected and suspended in 80 μL triton-X-100 (1% in PBS) to obtain cell lysates by twice freeze-thaw. Then the supernatant was collected after centrifugation at 9391 G for 10 min for subsequent analysis. Finally, 50 μL of supernatant was separately mixed evenly with 50 μL ATP working solution and the mixture was incubated for 30 min at room temperature. The absorbance at 570 nm was monitored with microplate reader.

### Cell apoptosis assay

Annexin V/PI staining was used to analyze the apoptosis of cancer cells. 4T1 cells were seeded into 6-well plates at a density of 10 × 10^4^ cells per well. The cells were permitted to resume exponential growth for 24 h and treated with various drugs. After another 24 h incubation, the supernatant was collected in 1.5 mL tubes. The cells were treated with 100 μL of 0.25% Trypsin-EDTA for 2 min, subsequently 300 μL fresh medium were added which was combined with the corresponding supernatant. The cells were obtained following centrifugation at 434 G for 5 min and washed twice with PBS, and then resuspended in 500 μL of binding buffer (1 ×). The cells were stained with Annexin V/Alexa Fluor 647 and PI (20 μg/mL) for 5 min at room temperature in the dark. The fluorescence signal was immediately detected by flow cytometry and the data were analyzed by FlowJo software.

### ROS generation assay

The generation of intracellular ROS within 4T1 cells was measured with DCFH-DA, BBoxiProbe O28 and BBoxiProbe O68 and evaluated by flow cytometry. 4T1 cells were seeded into 6-well plates at a density of 10 × 10^4^ cells per well and cultured at 37 °C for 24 h. The cells were treated with rhein (6.25 μM), DCA (6.25 μM), R + D (6.25 μM + 6.25 μM) and Rhein-DCA (6.25 μM) for another 6 h. Cells were detached from the plates with 0.25% Trypsin-EDTA after washed twice with PBS, flowing staining with DCFH-DA (10 μM), BBoxiProbe O28 (Ex = 516 nm, Em = 606 nm) and BBoxiProbe O68 (Ex = 516 nm, Em = 606 nm) for 20 min at 37 °C in the dark.

The level of intracellular superoxide radical anion in 4T1 cells was analyzed with DHE by confocal microscope. 4T1 cells were seeded into confocal dishes with a density of 10 × 10^4^ cells per well and treated with various drugs for 24 h and washed twice with PBS, flowing staining with DHE probe (5 μM) for 30 min at 37 °C in the dark. The cellular nuclei were stained with Hoechst 33342 for 10 min at room temperature. The fluorescent images were observed by confocal microscope.

### Immunogenic cell death detection* in vitro*

4T1 cells were seeded in 6-well plates and confocal dishes with an identical density of 10 × 10^4^ cells per well for 24 h cultivation. The cells were processed with indicated drugs at a concentration of 6.25 μM for another 24 h. The cells were washed twice with PBS and then fixed with 4%-paraformaldehyde (1 mL) for 15 min at 37 ^o^C, followed by two washes with PBS. (1) The cells on confocal dishes were next permeabilized with triton-X-100 for 15 min at room temperature, blocked with BSA solution (5% in PBS) for 1 h at room temperature, and further incubated with rabbit anti-mouse HMGB-1 (1:500) overnight at 4 ^o^C. Afterwards, the cells were incubated with secondary antibody (1:500) for 1 h at room temperature, washed with PBS, and then stained with Hoechst 33342 (10 μg/mL) for 10 min in the dark at room temperature. Finally, confocal microscope was employed to observe the HMGB1 protein within the cells. The secretion of HMGB1 in culture medium was detected by ELISA assay. The culture media was collected and centrifuged at 1000 G for 20 min, the supernatant was collected and detected with ELISA kit according to the manufacturer's protocol. (2) The cells in the 6-well plates were collected in 1.5 mL tubes, stained with rabbit anti-mouse calreticulin (1:1000) for 30 min at 4 ^o^C, washed twice with PBS, and then analyzed with flow cytometry. (3) For the extracellular ATP content, the cells were cultured for 6, 12 and 24 h at 37 ^o^C, and the medium were collected and centrifuged at 1000 G for 20 min, the supernatant was collected and detection with ELISA kit according to the manufacturer's protocol.

### Stability of Rhein-DCA in gastrointestinal environment

Prepare phosphate buffer solutions at pH 1.5 (gastric simulation) and pH 6.8 (intestinal simulation). Rhein-DCA was added to the buffer and incubated at 37°C. Samples were collected at 0, 2, 4, and 6 h for HPLC analysis. Calculate stability (%) = (C_t_ / C_0_) × 100, where C_t_ and C_0_ are concentrations at time t h and 0 h, respectively.

### *In vivo* experiments

Balb/c female mice (6 weeks) and Sprague-Dawley (SD) rats were purchased from Hubei provincial laboratory animal research center (Wuhan, P. R. China). All related animal experiments were approved by the Animal Welfare Committee of the Animal Experiment Center of Wuhan University. All Balb/c mice were shaved two days in advance and then subcutaneously injected with 4T1 cells (5 × 10^6^ cells in 50 μL of PBS per mouse) into the back of the right leg. The 4T1 tumor bearing mice were randomly divided into five groups (5 mice per group) which containing vehicle, rhein, DCA, R + D and Rhein-DCA groups when the tumor volumes reached about 100~120 mm^3^. The tumor size was calculated with the following formula:

V = L × W^2^ / 2

where *V* (mm^3^) was the volume of the tumor, *L* (mm) and *W* (mm) were the length and width of the tumor, respectively.

Subsequently, mice were orally treated with vehicle (2.85% anhydrous ethanol / 10% cremophor / 0.02% HCl (0.1 M) / 87.15% saline) and rhein, DCA, R + D and Rhein-DCA (20 mg/kg, suspended in 2.85% anhydrous ethanol / 10% cremophor / 0.02% HCl (0.1 M) / 87.15% saline) every day. The body weight and tumor size changes of each mouse were measured and recorded every two days.

After 24 days, the mice were euthanized, and then tumors were excised to photographing and weighting. The tumors in each group were performed with TUNEL (green fluorescence) and H&E assay. In addition, immunohistochemical staining was conducted on the tumor tissue to determine the pPDHA1-Ser^293^ protein level. Besides, immunofluorescence staining was used to detect the expression of HMGB1 protein in the tumor.

The major organs (heart, liver, spleen, lung and kidney) were also excised for photographing and H&E staining. The blood biochemistry indexes, including ALT, AST, CREA and UREA were tested.

### Lactate detection in tumor tissue

Tumor tissues were collected and homogenized in ice-cold PBS (1:10, w/v). The tissue was disrupted using sonication in ice bath (power 400 W, the duration of sonication is 3 s, the interval duration of 3 s, total sonication time of 20 min). The homogenate was centrifuged at 5000 G for 10 min at 4 °C to obtain the supernatant. The lactate content was analyzed with a lactate ELISA kit according to the manufacturer's protocol. The results were normalized to the weight of the tumor and reported as µmol lactate/g tissue.

### Tumor-associated macrophages detecting

The macrophages within the tumor microenvironment of tumor tissues were further analyzed. Tumor tissues were minced into small pieces and then digested in a solution containing 1 mg/mL of collagenase IV, 0.1 mg/mL of hyaluronidase and 0.1 mg/mL of DNAase I at 37 °C for 1.5 h. The supernatants containing single cells were collected by filtering through 200 mesh filters. Cells were obtained by centrifugation and further blocked with anti-CD16/32 (1 μg/100 μL) for 10 min in an ice bath. Thereafter, FITC anti-mouse CD11b (1 μg/100 μL) and PE anti-mouse CD80 (1 μg/100 μL) antibodies were employed to stain the cells in an ice bath for 20 min. After that, the cells were collected and fixed with 4%-paraformaldehyde at room temperature for 20 min, and then stained with APC anti-mouse CD206 antibody (0.5 μg/100 μL, in 1 × Fix/Perm buffer) at room temperature in the dark for 20 min. Finally, cells were analyzed with flow cytometry after being resuspended in 1 × Perm/Wash buffer.

### Pharmacokinetic study and tissue distribution

HPLC was used to evaluate the absorption and distribution of Rhein-DCA in mice after oral administration. Rhein-DCA was orally administered to Sprague-Dawley (SD) rats at 20 mg/kg. and the blood samples were serially collected at predefined time points (0.5, 1.5, 2, 3, 4, 5, 6, 8, 10 and 24 h). Centrifuge the blood at 3000 G for 10 min to separate serum, and the serum was mixed with methanol (1:3, v/v), vortexed for 3 min, and centrifuged at 13000 G for 10 min. The supernatant was filtered through a 0.22 µm membrane for HPLC assay. Pharmacokinetic parameters were derived using DAS 2.0 software with non-compartmental analysis (NCA).

4T1-bearing Balb/c mice were orally administered with Rhein-DCA (20 mg/kg), major organs (heart, liver, spleen, lung, kidney) and tumor tissues were harvested and weighed at 2, 6, and 24 h after administration. Each organ was homogenized with sonication in ice bath (power 400 W, the duration of sonication is 3 s, the interval duration of 3 s, total sonication time of 20 min). Centrifuge the homogenate at 13000 G for 10 min at 4°C to obtain the supernatant for HPLC.

### Statistical analysis

Statistical analysis was performed by a student's *t-test* with GraphPad at a significance level of *p* < 0.05(*), *p* < 0.01(**), *p* < 0.001(***) and *p* < 0.0001(****).

## Supplementary Material

Supplementary methods, figures and table.

## Figures and Tables

**Table 1:** Reagent concentrations

PDK1 enzyme	PDK1 tide	ATP	Compounds
50 ng/well	0.2 μg/μL	5 μM	0.08, 0.4, 2 μM

## Abbreviations

ALT: alanine aminotransferase
AST: aspartate aminotransferase
ATP: adenosine triphosphate
UREA: urea
CLSM: confocal laser scanning microscope
CREA: creatinine
CRT: calreticulin
CsA: cyclosporin A
DAMPs: damage-associated molecular patterns
DCA: dichloroacetate
DCFH-DA: 2',7'-dichlorofluorescein diacetate
DEGs: differentially expressed genes
DHE: dihydroethidium
DMSO: dimethyl sulfoxide
EG: ethylene glycol
ES: enrichment scores
ETC: electron transport chain
GO: gene ontology
GSEA: gene set enrichment analysis
H&E: hematoxylin-eosin
HMGB1: high mobility group box 1
HPLC: high performance liquid chromatography
ICD: immunogenic cell death
IF: immunofluorescence
IHC: immunohistochemical
KEGG: kyoto encyclopedia of genes and genomes
mPTP: mitochondrial permeability transition pore
MTT: 3-(4,5)-dimethylthiahiazo(-z-y1)-3,5-di-phenytetrazoliumromide
Δψm: mitochondrial membrane potential
^1^O₂: singlet oxygen
·OH: hydroxyl radicals
OCR: oxygen consumption rate
OXPHOS: oxidative phosphorylation
PDH: pyruvate dehydrogenase complex
PDK: pyruvate dehydrogenase kinase
R + D: mixture of rhein and DCA
ROS: reactive oxygen species
TAMs: tumor-associated macrophages
TCA: tricarboxylic acid cycle
TME: tumor microenvironment
TUNEL: TdT-mediated dUTP nick-end labeling
