# Supplementary material for: Mitochondria-targeting natural product rhein conjugated with dichloroacetate as the dual inhibitor of glycolysis and oxidative phosphorylation to off energize cancer cells and induce ROS storm
Source: Theranostics. 2025 Mar 31;15(11):4909–29. doi: 10.7150/thno.107812 (PMC12036875; doi:10.7150/thno.107812)
Supplement: Supplementary file 1 — Supplementary methods, figures and table. [file thnov15p4909s1.pdf]

Mitochondria-targeting natural product rhein conjugated with dichloroacetate as the dual inhibitor of glycolysis and oxidative phosphorylation to off energize cancer cells and induce ROS storm

Zhili Zhang[1], Shiming Tang[1], Minghui Qi[1], Hongyang Zhao[1], Meng Wu[2,*], Shi-Wen Huang[1,2,*]

1. Key Laboratory of Biomedical Polymers of Ministry of Education, Department of Chemistry, Wuhan University, Wuhan 430072, P. R. China
2. Department of Ultrasound, Zhongnan Hospital of Wuhan University, Wuhan University, Wuhan 430071, P. R. China

[*]Corresponding authors: Prof. Meng Wu, Department of Ultrasound, Zhongnan Hospital of Wuhan University, Wuhan University, Wuhan 430071, P. R. China. **E-mail:** wb000713@whu.edu.cn. Prof. Shi-Wen Huang, Key Laboratory of Biomedical Polymers of Ministry of Education, Department of Chemistry, Wuhan University, Wuhan 430072, P. R. China. **E-mail:** swhuang@whu.edu.cn

Materials and reagents

Rhein (98%) and Phosphoric acid (HPLC grade) was purchased from Macklin Inc (Shanghai, P. R. China). Ethylene glycol (EG, 99%) was purchased from Energy Chemical Inc (Anhui, P. R. China). Dichloroacetyl chloride (> 98%) was purchased from TCI Development Co., Ltd (Shanghai, P. R. China). Triethylamine (99.7%) was purchased from Acros Organics Co., Ltd (Belgium). Sodium 2,2-dichloroacetate (DCA-Na, 97%) was purchased from Bidepharm Inc (Shanghai, P. R. China). Dimethyl sulfoxide (DMSO, AR), Ethanol (≥ 99.8%), Methanol (≥ 99.5%), Dichloromethane (CH_2Cl_2, ≥ 99.5%), Sodium hydrogen carbonate ($NaHCO_3$, AR), Sulfuric acid (H_2SO_4, GR), Hydrochloric acid (HCl, GR) and Sodium sulfate ($NaSO_4$, AR) were purchased from Sinopharm Chemical Reagent Co., Ltd (Shanghai, P. R. China). Cyclosporin A (CsA) was purchased from Aladdin Chemical Co. Ltd. (Shanghai, P. R. China). Phosphate Buffered Saline (PBS) was purchased from Dingguo Changsheng Biotechnology Co., Ltd (Beijing, P. R. China). Castor oil ethoxylated (pH-range: 6.0-8.1) was purchased from InnoChem Science & Technology Co., Ltd (Beijing, P. R. China). Fetal bovine serum (FBS) was purchased from Cell-Box Biological Products Trading Co., Ltd (HK, P. R. China). Methanol (HPLC grade), 0.25% Trypsin-EDTA and Penicillin streptomycin were purchased from Thermo Fisher Scientific Inc (USA). Roswell Park Memorial Institute-1640 (RPMI-1640) and Dulbecco'S Modified Eagle Medium (DMEM) were purchased from Procell Life Science&Technology Co.,Ltd (Wuhan, P. R. China). Thiazolyl blue tetrazolium bromide (MTT) (≥ 98%), Lactic Acid (LA) Content Assay Kit and Reactive Oxygen Species Kit (DHE) were purchased from Solarbio Science&Technology Co., Ltd (Beijing, P. R. China). Reactive Oxygen Species Assay Kit (DCFH-DA) and Bovine Serum Albumin, Fraction V (BSA) were purchased from Gen-View Scientific Inc (USA). AnnexinV Alexa Fluor 647/PI Apoptosis Detection Kit was purchased from 4A Biotech Inc (Suzhou, P. R. China). MitoTracker® Deep Red FM and Hoechst 33342 were purchased from Yeasen Biotechnology Co., Ltd (Shanghai, P. R. China). Enhanced Mitochondrial Membrane Potential Assay Kit with JC-1, Mitochondrial Membrane Potential Assay Kit with TMRE, Mitochondrial Permeability Transition Pore (mPTP) Assay Kit, ER-Tracker Red, Lyso-Tracker Red, Enhanced BCA Protein Assay Kit, Hoechst 33342 Staining Solution and Triton X-100 were purchased from Beyotime Biotech. Inc (Shanghai, P. R. China). Singlet Oxygen Detection Kit (Red Fluorescence) and Hydroxyl Radical Detection Kit (Red Fluorescence) were purchased from BestBio Biotechnology Co., Ltd (Shanghai, P. R. China). PhosphoWorks™ Colorimetric ATP Assay Kit was purchased from AAT Bioquest Co., Ltd (USA). Mouse ATP ELISA Kit was purchased from Mskbio Biotechnology Co., Ltd. (Wuhan, P. R. China). PDK1 Kinase Assay and ADP-Glo™ Kinase Assay were purchased from Promega Biotech Co., Ltd (USA). Seahorse XF assay media, Seahorse XF Cell Mito Stress Test Kit were purchased from Agilent Technologies, Inc (USA). 4%-paraformaldehyde was purchased from Labgic Technology Co., Ltd (Anhui, P. R. China). Mouse Lactate ELISA Kit was purchased from Meike Biotechnology Co., Ltd. (Jiangsu, P. R. China). Mouse HMGB1 (High mobility group protein B1) ELISA Kit was purchased from Elabscience Biotechnology Co., Ltd. (Wuhan, P. R. China). Rabbit anti-mouse calreticulin (SPC-122B-FITC) was purchased from StressMarq Biosciences Inc (USA), Rabbit anti-mouse HMGB-1 (651403), APC anti-mouse CD206Antibody (141708) and FITC anti-mouse/human CD11b Antibody (101206) were purchased from BioLegend, Inc (USA). BD PharmingenTM PE Hamster Anti-Mouse CD80 (553769) was purchased from BD Biosciences Inc (USA). HRP conjugated Goat Anti-Rabbit IgG (H+L) (AS1107) was purchased from Aspen Biotechnology Co., Ltd. ECL Chemiluminescent Substrate Kit and RIPA Total Protein Extraction Solution and were purchased from Aspen Biotechnology Co., Ltd and Servicebio Technology Co., Ltd (Wuhan, P. R. China). 50 × Cocktail protease inhibitor, Phosphatase inhibitor, Phenylmethanesulfonyl fluoride (PMSF), super ECL detection reagent, polyvinylidene fluoride membranes (PVDF membranes, 0.45 μm), Anti-β Actin Rabbit pAb (GB11001), Anti-Cytochrome C Rabbit pAb (GB11080), HRP conjugated Goat Anti-Rabbit IgG (H+L) (GB23303) were purchased from Servicebio Technology Co., Ltd (Wuhan, P. R. China). β-actin Rabbit pAb (TDY051) was purchased from TDY Biotech Co., Ltd (Beijing, P. R. China). Anti-PDHA1 (phosphor S^{293}) antibody (ab177461) was purchased from Abcam (UK).

Instruments and characterization

^{1}H and ^{13}C NMR spectra were obtained with AVANCE III HD 400MHz Nuclear Magnetic Resonance Spectrometer (NMR), (Bruker, Switzerland). HRMS spectra were performed with TripleQTOF5600 + liquid Chromatography High Resolution Mass Spectrometer (HRMS), (Absciex, USA). The UV/Vis absorption spectra were measured with Lambda35 Ultraviolet-visible Spectrophotometer (Perkin-Elmer, USA). Fluorescence emission spectra were measured with RF-5301PC Fluorescence Spectrophotometer (Shimadzu, Japan). Fourier Transform-Infrared (FT-IR) spectra were obtained with FTS 6000 Spectrometer (Bio-Rad, USA). Shimadzu HPLC (LC-15CT, Japan). Ultrasonic Cell Crusher SCIENTZ-IID (Ningbo Scientz, P. R. China). Confocal laser scanning microscope images were collected with Ultraview VOX Spinning Disc Confocal Microscope (Perkin-Elmer, USA). Flow cytometry were conducted with CytoFlex flow Cytometer (Beckman, USA). Absorbance properties were obtained with Model 550 Microplate Reader (Bio-Rad, USA). Luminescent properties were obtained with SpectraMax iD5 Multi-Mode Microplate Reader (Molecular Devices, USA). Oxygen Consumption Rate (OCR) were measured with Seahorse XFe24 (Agilent, USA).

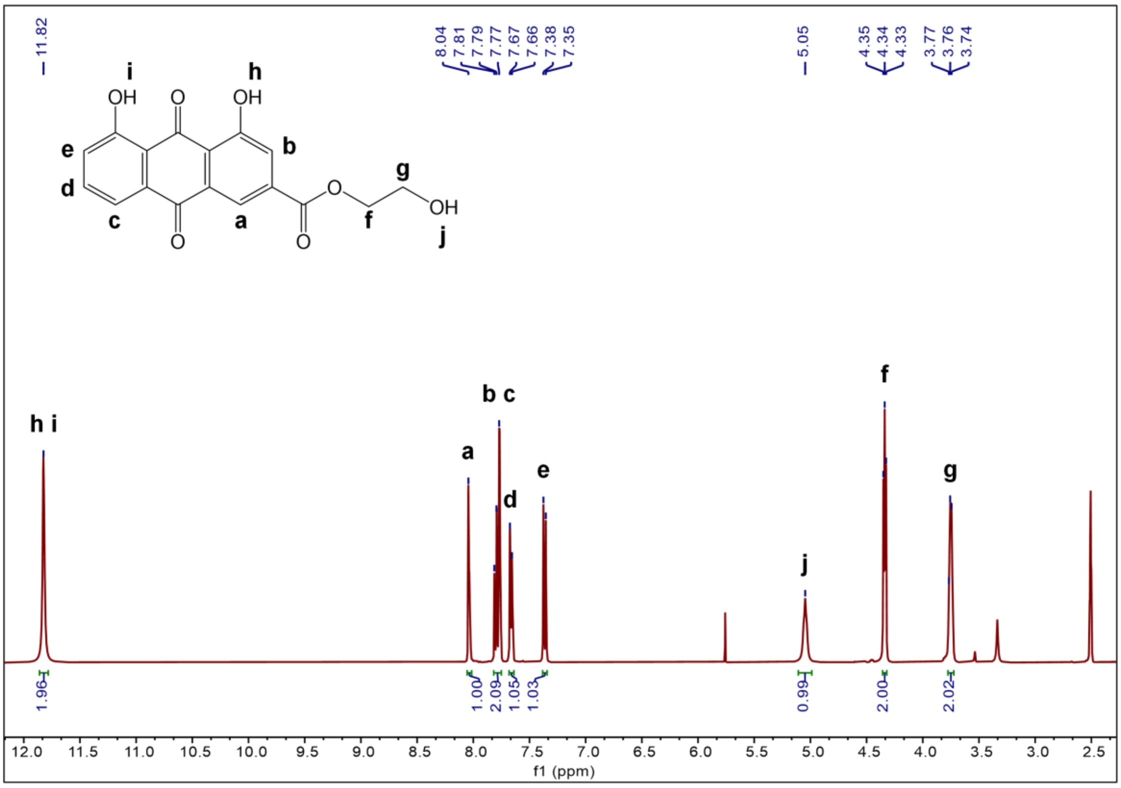

Figure S1. ^{1}H-NMR spectrum (400 MHz, DMSO-d6) of Rhein-EG.

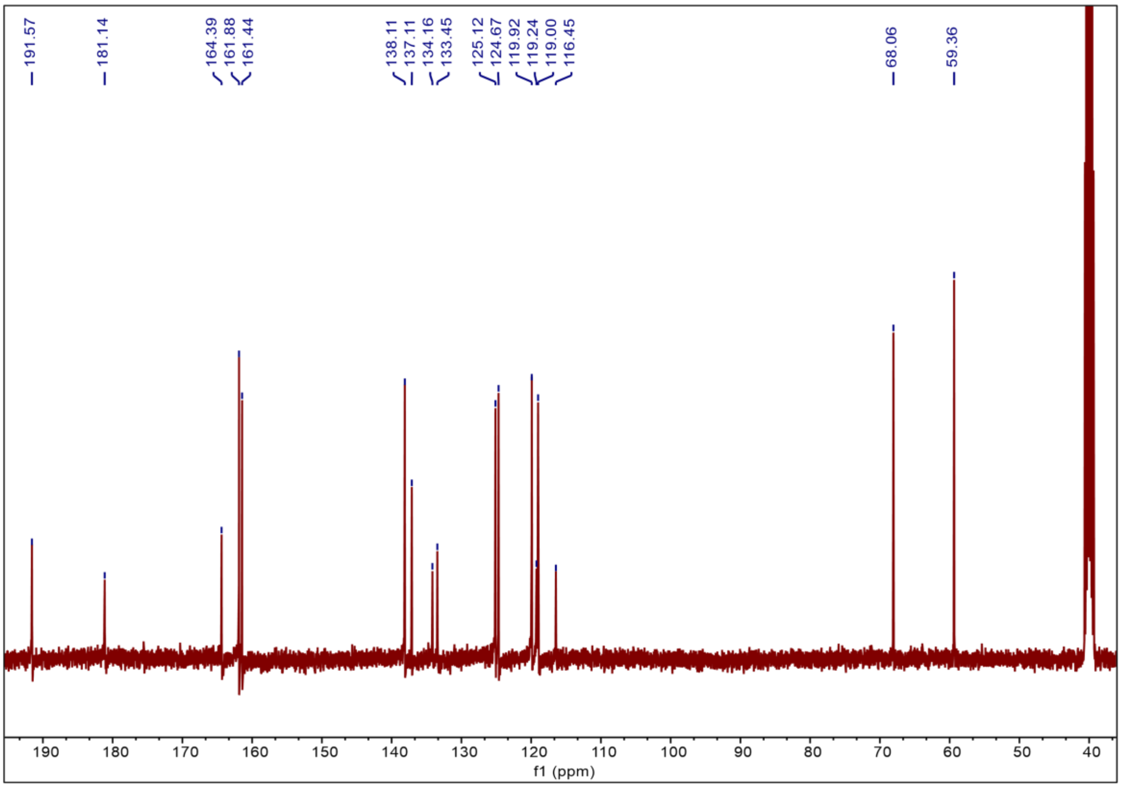

Figure S2. ^{13}C-NMR spectrum (400 MHz, DMSO-d6) of Rhein-EG.

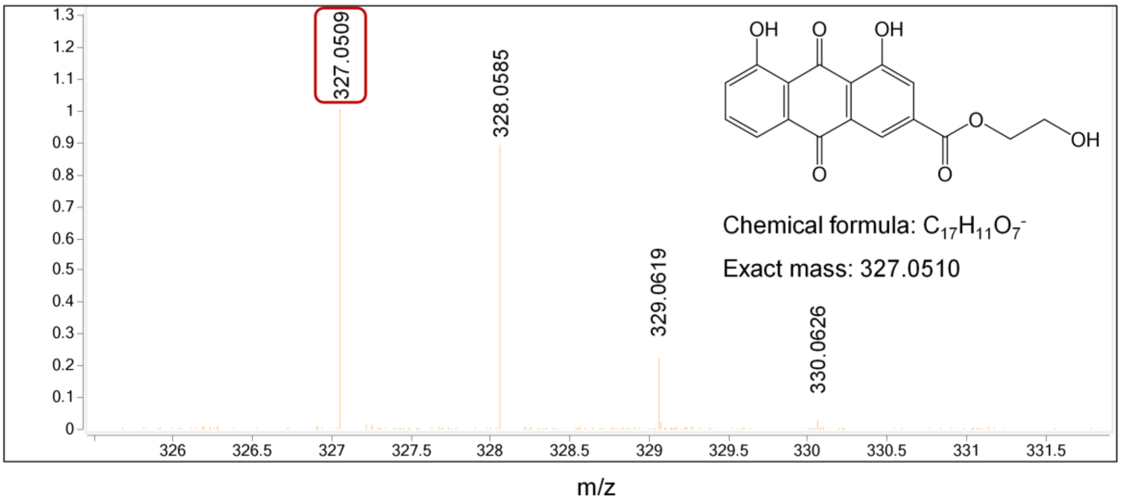

Figure S3. HRMS spectrum (CH_3OH) of Rhein-EG.

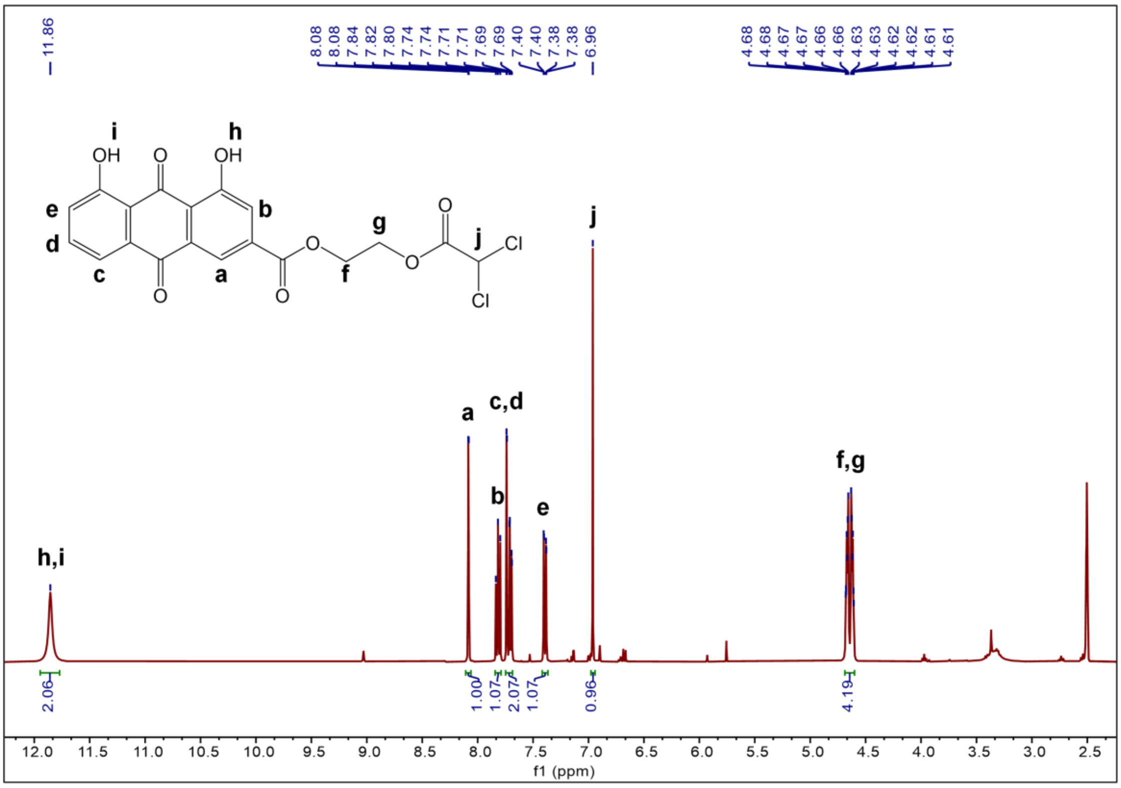

Figure S4. ^{1}H-NMR spectrum (400 MHz, DMSO-d6) of Rhein-DCA conjugate.

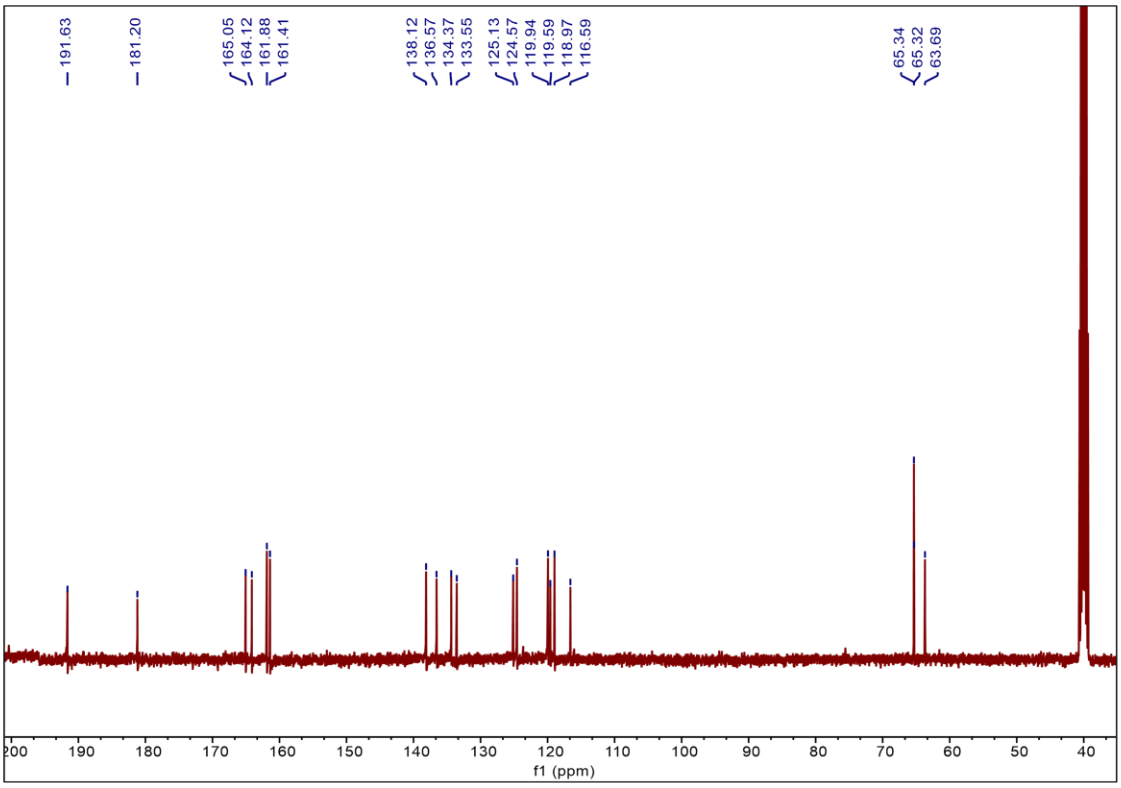

Figure S5. ^{13}C-NMR spectrum (400 MHz, DMSO-d6) of Rhein-DCA conjugate.

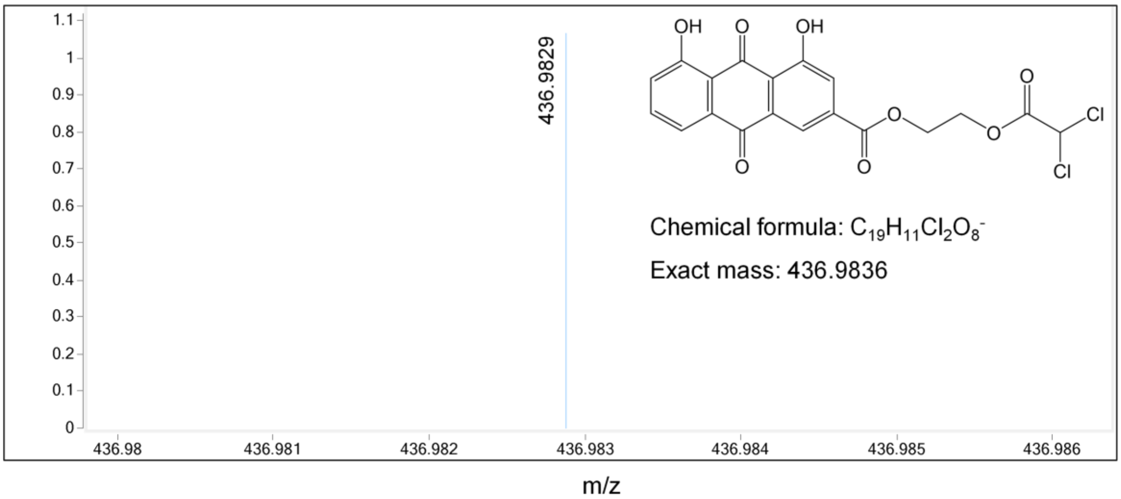

Figure S6. HRMS spectrum (CH_3OH) of Rhein-DCA conjugate.

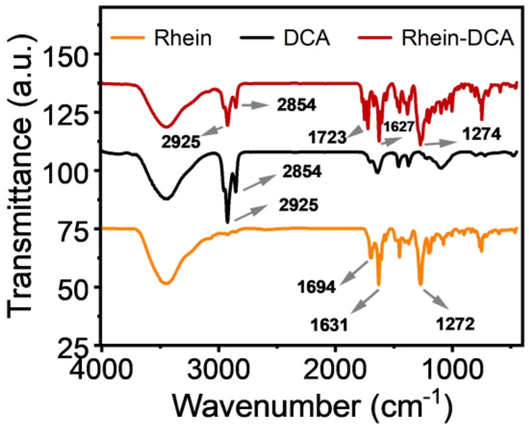

Figure S7. The fourier transform-infrared (FT-IR) spectrum of rhein, DCA and Rhein-DCA conjugate. For DCA, the vibration located at 2925 and 2854 cm^{-1} is attributed to C-H stretching. The spectrum of rhein showed a C-O stretching vibration at 1272 cm^{-1}, and C=O stretching vibration at 1631 and 1694 cm^{-1}. The presence of the peak of C-H stretch at 2925, 2854 cm^{-1} and C-O stretch at 1274 cm^{-1}, simultaneously accompanied by the absorption band characteristic of ester group at 1723 cm^{-1}, indicating the formation of Rhein-DCA conjugate.

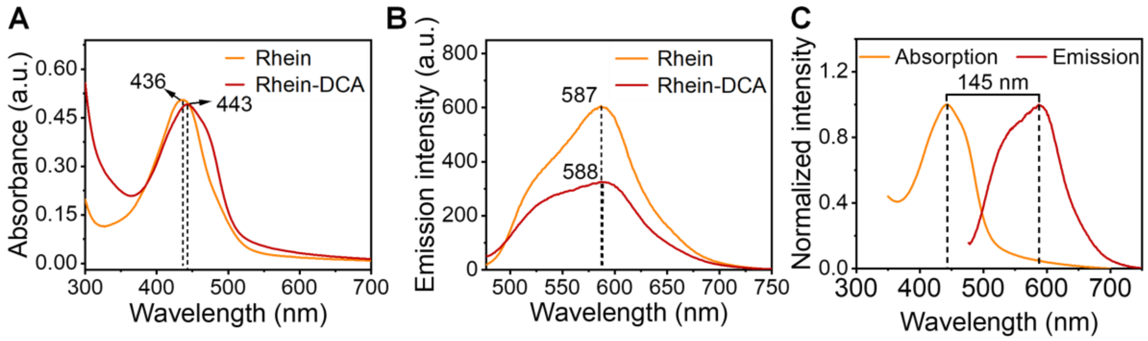

Figure S8. A) The UV/Vis absorption spectra displayed the characteristic anthraquinone group peak of rhein in Rhein-DCA conjugate. B) Fluorescence emission spectra (λex = 443 nm, monitoring the emission from 450 to 750 nm) of rhein and Rhein-DCA conjugate. The emission peak of rhein and Rhein-DCA conjugate were at 587 nm and 588 nm. C) The considerable stokes shift, amounting to 145 nm, and small degree of overlap between the normalized absorption and emission spectra of Rhein-DCA conjugate implied its potential in biological imaging.

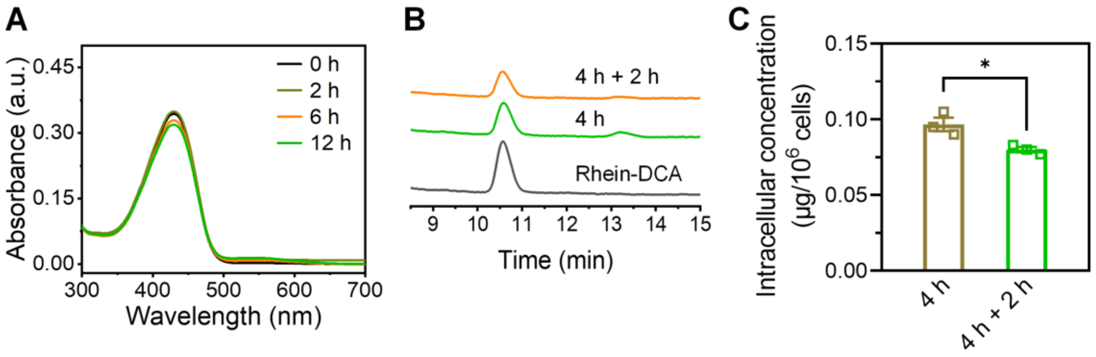

Figure S9. Stability of Rhein-DCA conjugate in A) PBS. B) The HPLC chromatogram of the Rhein-DCA content in 4T1 cells. C) The quantitative analysis of Rhein-DCA within cells based on the HPLC data. Data represent mean ± S.D., n = 3, $p^* < 0.05$.

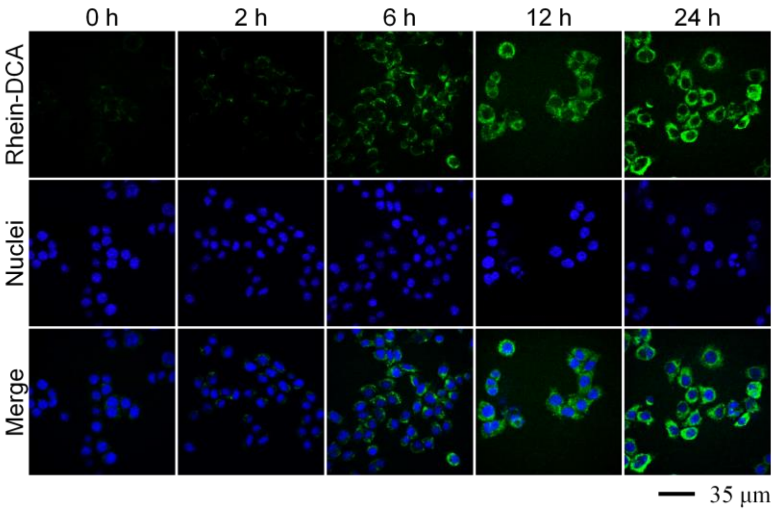

Figure S10. CLSM images of 4T1 cells treated with Rhein-DCA (6.25 μM) for 0, 2, 6, 12 and 24 h. Scale bar represents 35 μm.

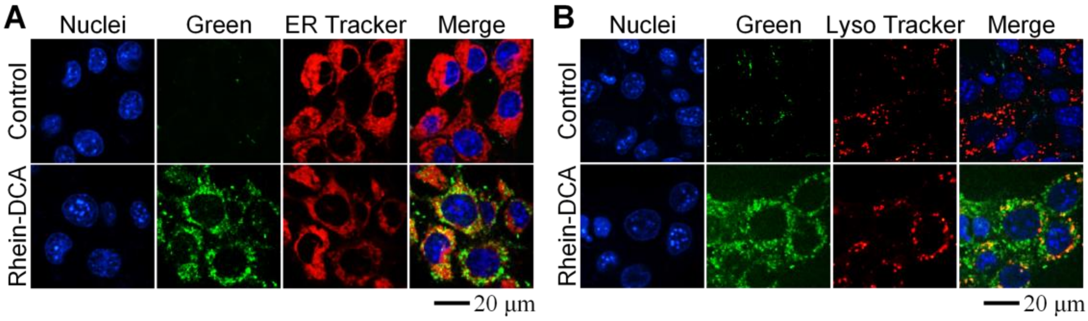

Figure S11. Confocal microscopy images of 4T1 cells treated with Rhein-DCA and then stained with A) ER-Tracker Red and B) Lyso-Tracker Red. Scale bar represents 20 μm.

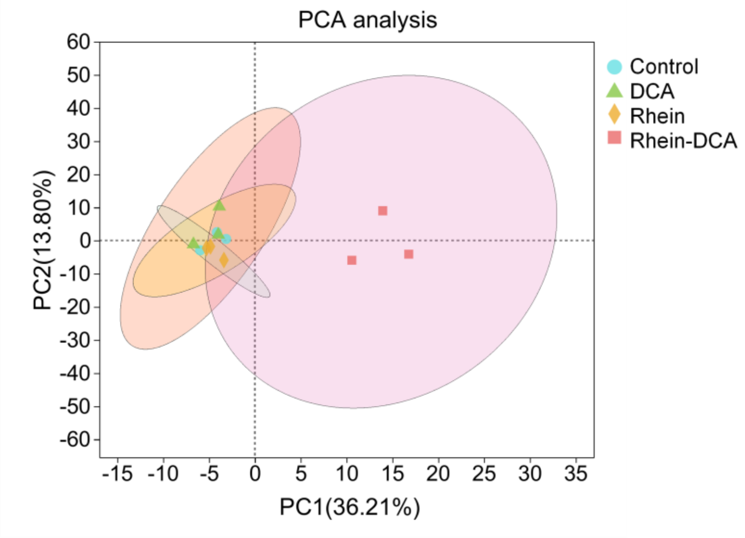

Figure S12. Principal coordinates analysis (PCA) of 4T1 cells in the control, DCA, rhein and Rhein-DCA groups.

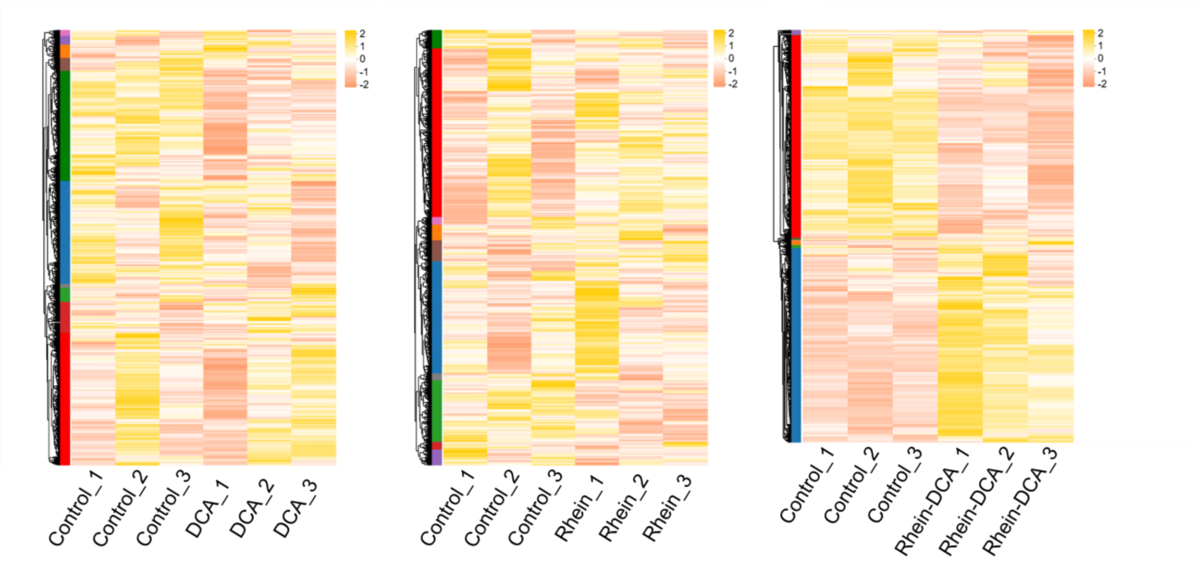

Figure S13. Comparison of whole genome cluster heat map of 4T1 cells treated with DCA (6.25 μM), rhein (6.25 μM), Rhein-DCA conjugate (6.25 μM) and nontreated cells (Control).

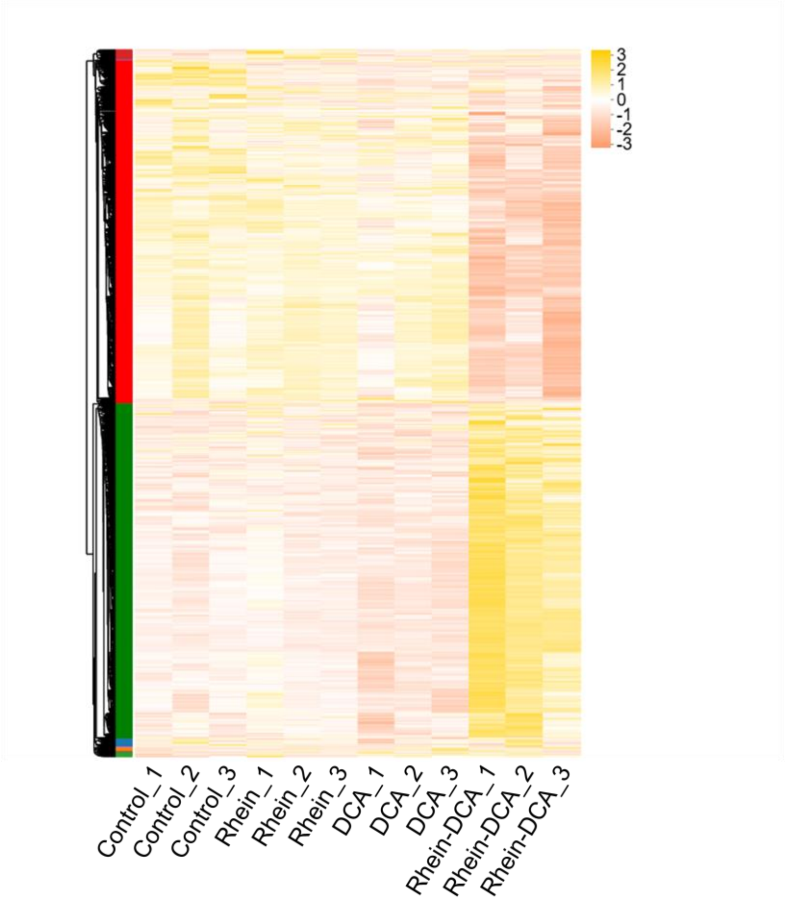

Figure S14. Gene expression heat map across different groups for all differential expression genes.

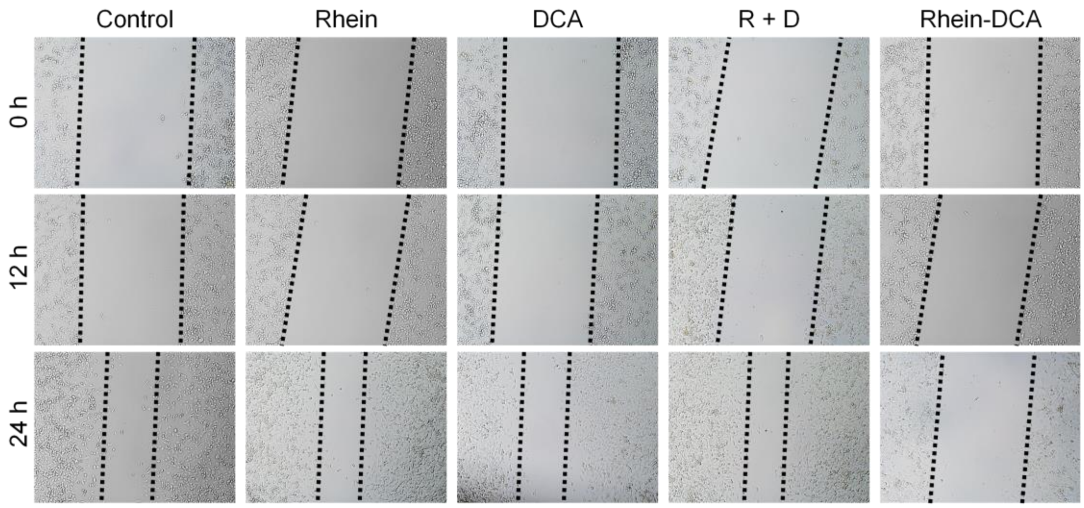

Figure S15. Migration and invasion evaluation of 4T1 cells after different treatment for 0, 12 and 24 h *via* scratch assay.

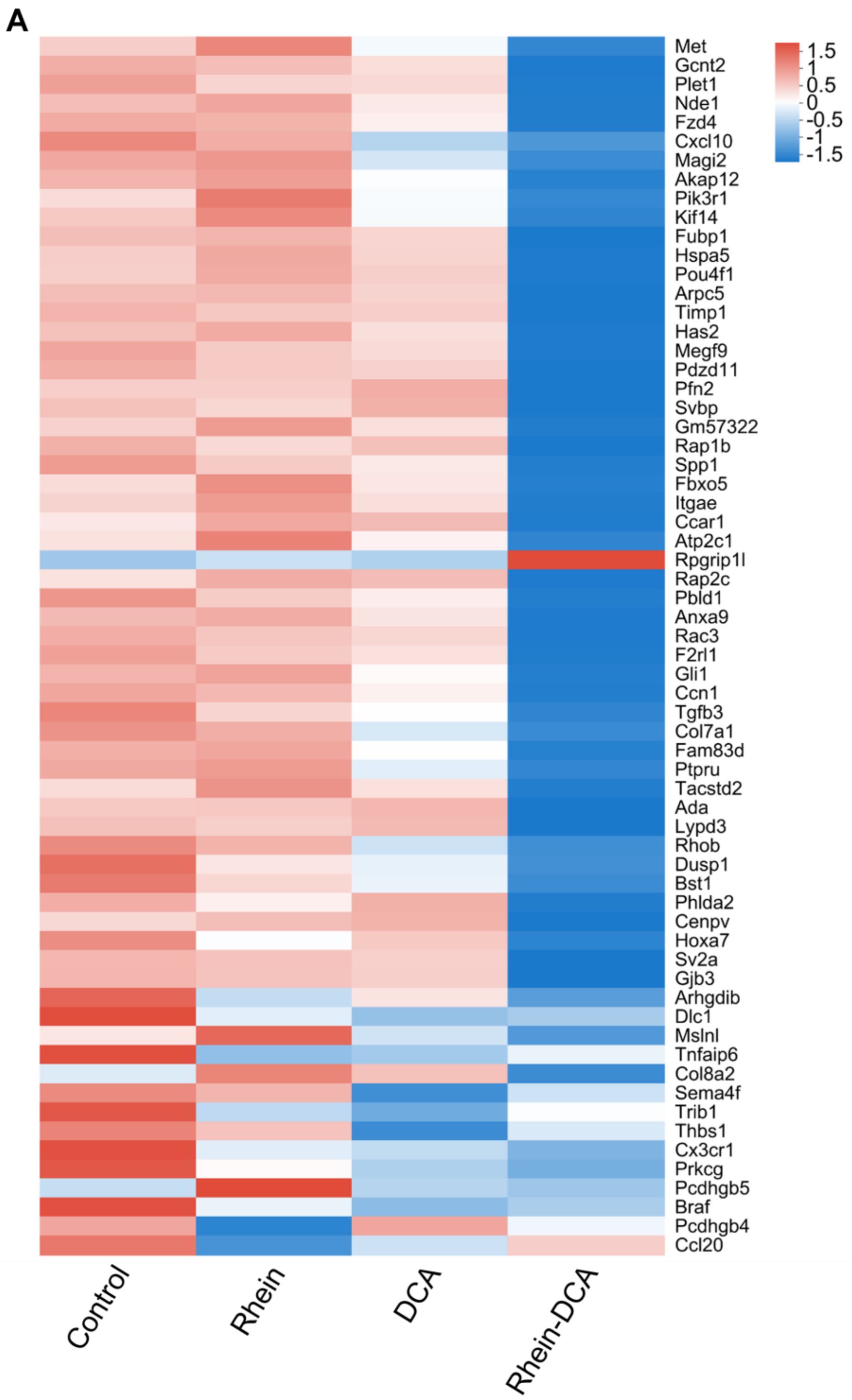

A
Met
Gcnt2
Plet1
Nde1
Fzd4
Cxcl10
Magi2
Akap12
Pik3r1
Kif14
Fubp1
Hspa5
Pou4f1
Arpc5
Timp1
Has2
Megf9
Pdzd11
Pfn2
Svbp
Gm57322
Rap1b
Spp1
Fbxo5
Itgae
Ccar1
Atp2c1
Rpgrip1l
Rap2c
Pbld1
Anxa9
Rac3
F2rl1
Gli1
Ccn1
Tgfb3
Col7a1
Fam83d
Ptpru
Tacstd2
Ada
Lypd3
Rhob
Dusp1
Bst1
Phlda2
Cenpv
Hoxa7
Sv2a
Gjb3
Arhgdib
Dlc1
Mslnl
Tnfaip6
Col8a2
Sema4f
Trib1
Thbs1
Cx3cr1
Prkcg
Pcdhgb5
Braf
Pcdhgb4
Ccl20
1.5
1
0.5
0
-0.5
-1
-1.5
Control
Rhein
DCA
Rhein-DCA

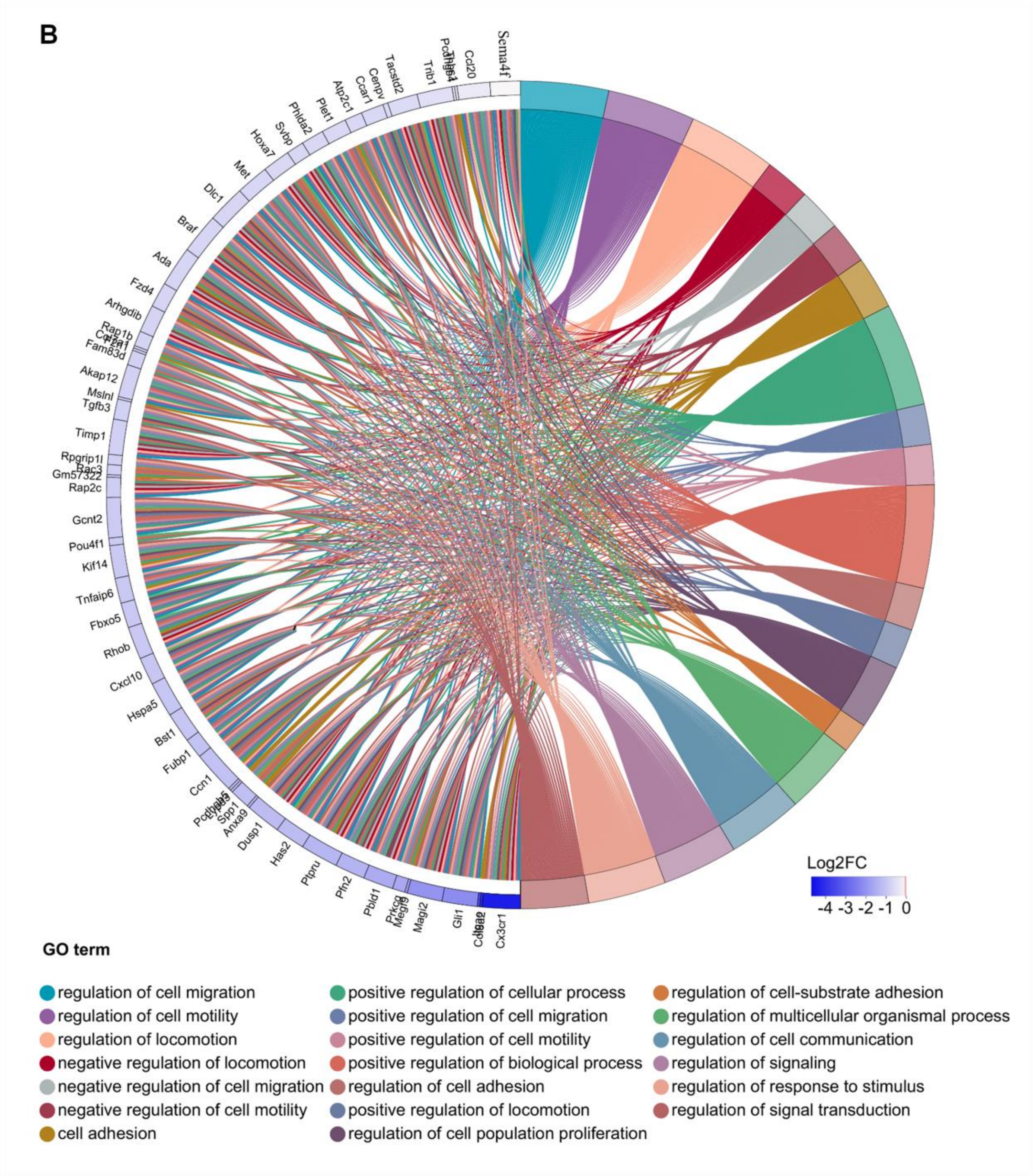

Figure S16. A) Gene cluster heatmap analysis of migration-related genes differentially expressed in 4T1 cells among control, rhein, DCA and Rhein-DCA-treated groups. B) GO analysis of downregulated migration-related pathways in 4T1 cells between Rhein-DCA group *vs* control group.

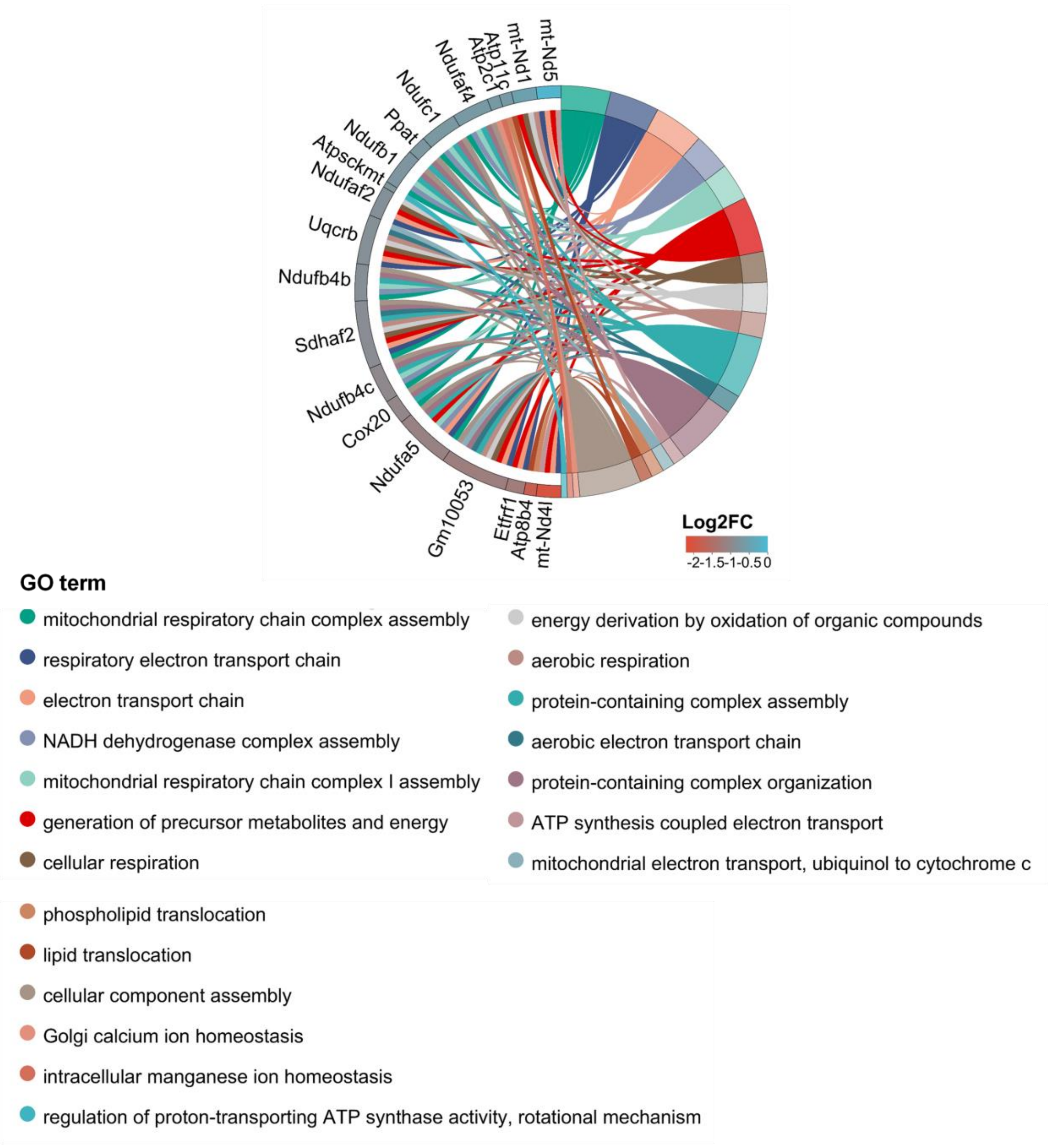

Figure S17. GO enrichment analysis of various genes related to OXPHOS pathway between Rhein-DCA *vs* control group.

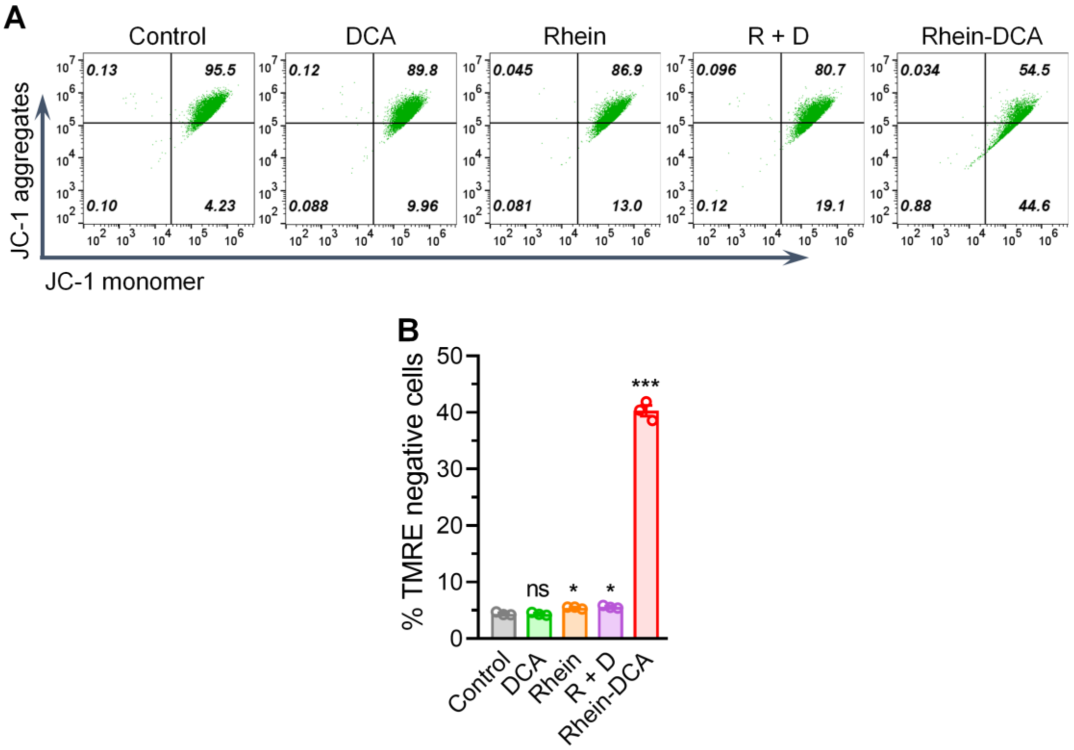

Figure S18. A) Flow cytometry analysis of 4T1 cells staining with JC-1 after different treatments for 24 h. B) Histogram plots show loss of TMRE staining in the PE channel. The graphed representation of the percentage of cells that lose TMRE (% TMRE negative cells) is shown to the degree of mitochondrial membrane depolarization. Data represent mean ± S.D., n = 3, $p^* < 0.05$, $p^{***} < 0.001$.

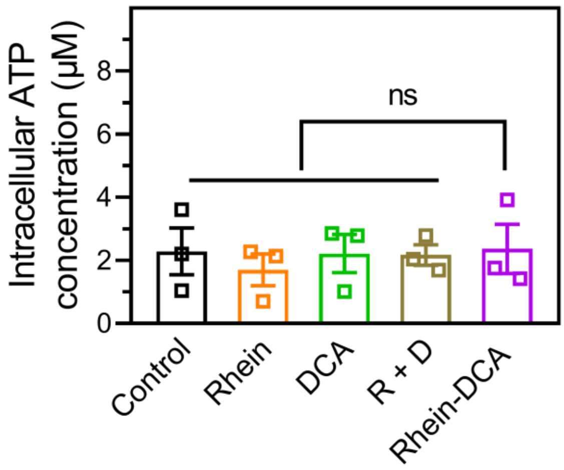

Figure S19. Intracellular ATP content in 293T cells after various treatments. Data represent mean ± S.D., n = 3.

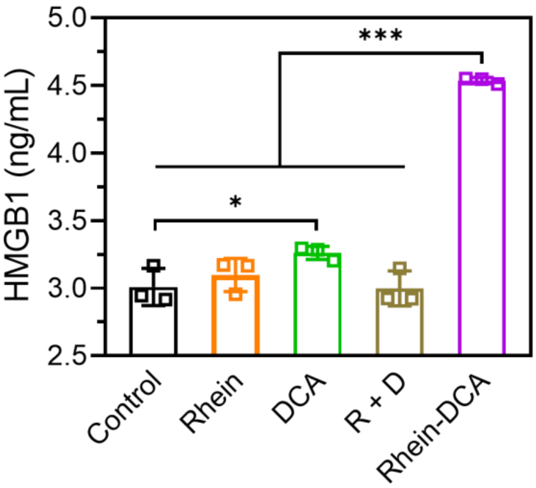

Figure S20. Extracellular release of HMGB1 after different treatments measured with an ELISA kit. Data represent mean ± S.D., n = 3, $p^* < 0.05$, $p^{***} < 0.001$.

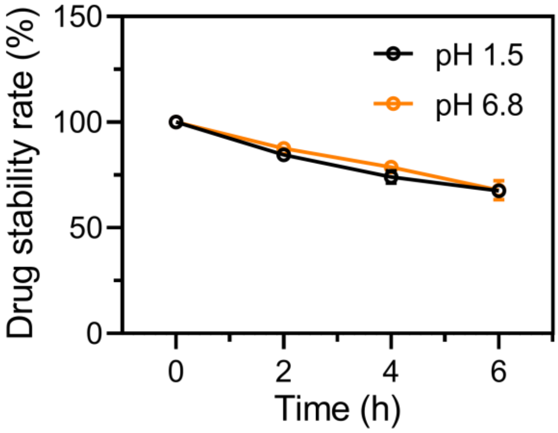

Figure S21. The percentage of Rhein-DCA remaining undegraded after incubation at different times under various pH values in simulated gastrointestinal environment. Data represent mean ± S.D., n = 3.

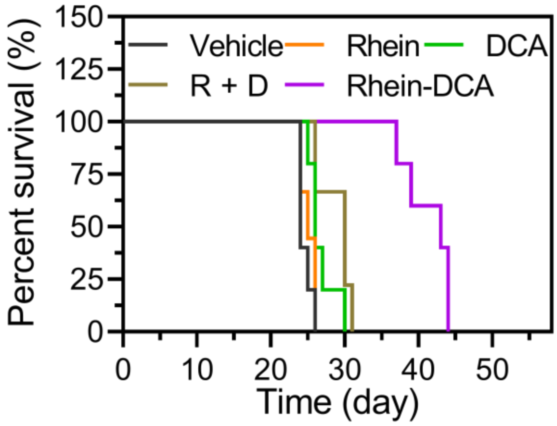

Figure S22. Survival curves for mice bearing 4T1 tumors (n = 5).

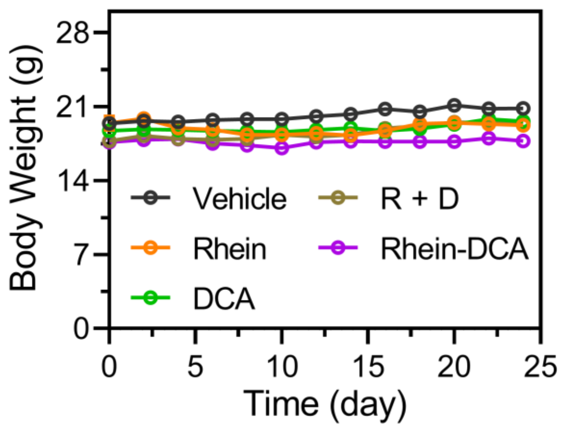

Figure S23. Body weight changes of mice during the treatment. Data represent mean ± S.D., n = 5.

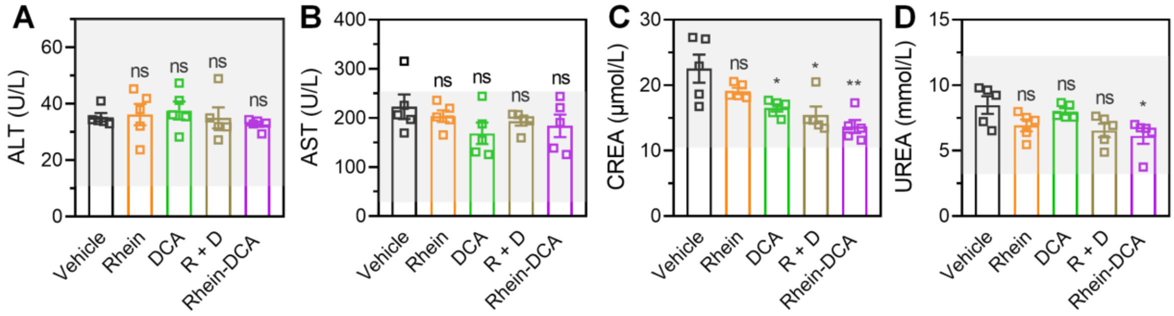

Figure S24. Blood biochemistry analysis of mice after various treatment for 24 days. The gray areas represent the normal range of normal mice in the laboratory. Data represent mean ± S.D., n = 5. $p* < 0.05$, $p** < 0.01$.

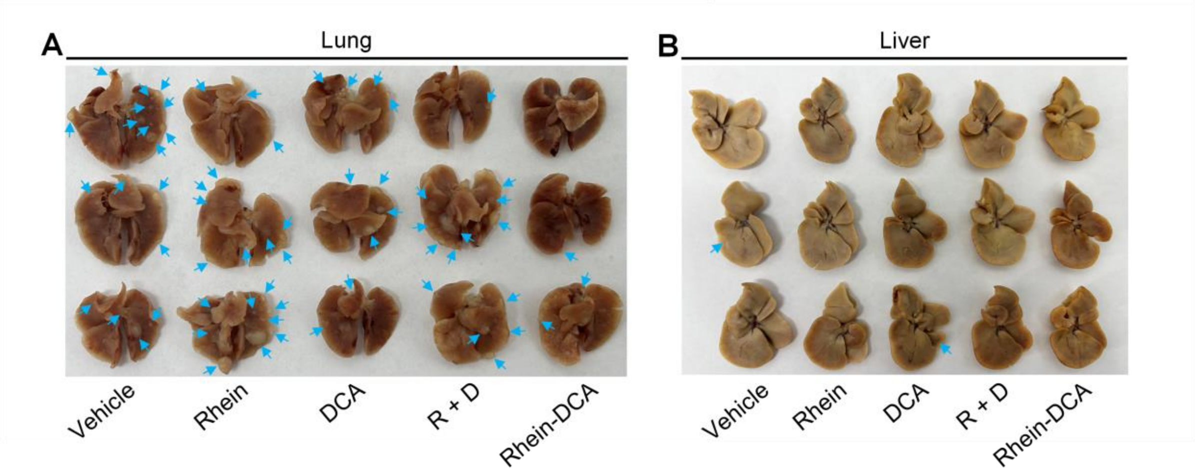

Figure S25. Representative photos of the whole lungs and livers of the mice after 24 days treatment.

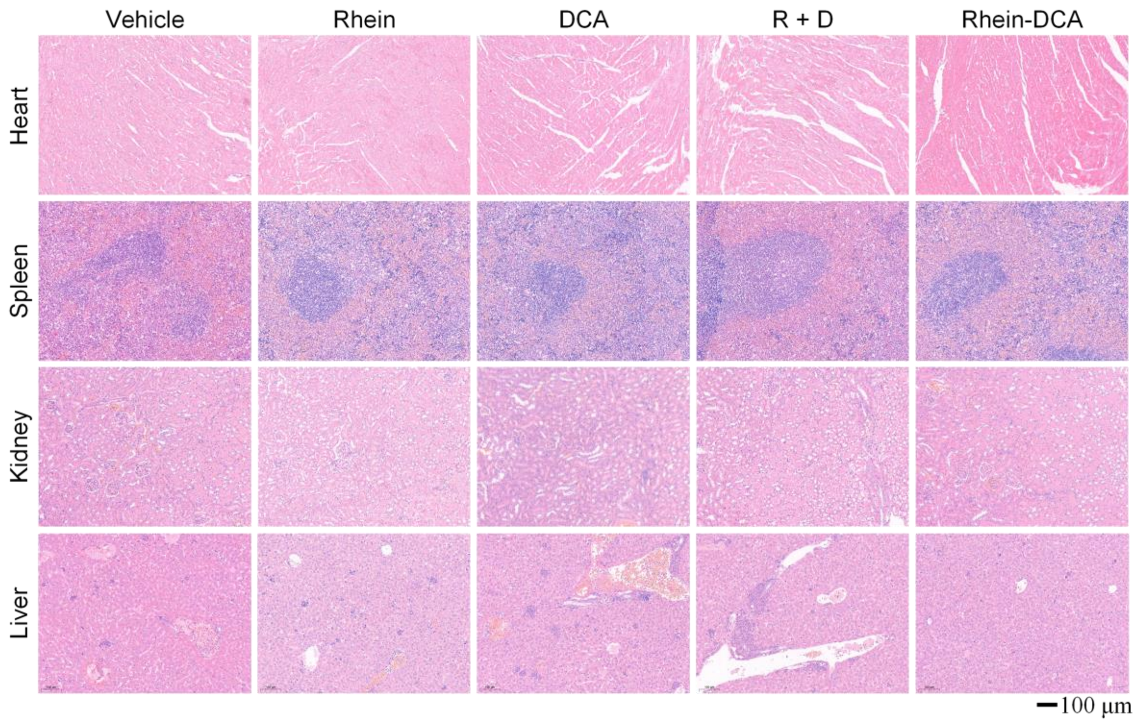

Figure S26. H&E staining images for the hearts, spleens, kidneys and livers of mice after 24 days treatment. Scale bar represents 100 μm.

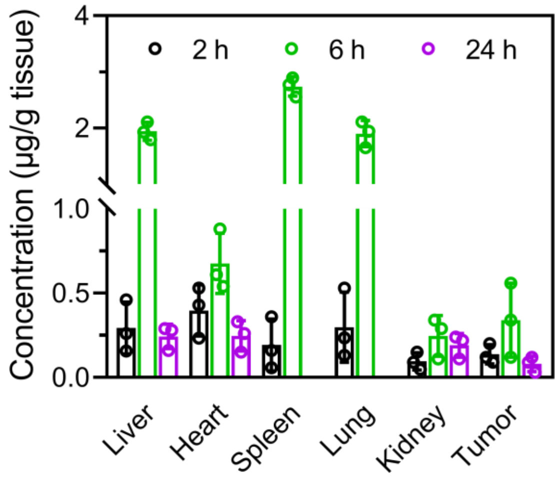

Figure S27. Distribution of Rhein-DCA in major organs of mice after treatment for 2, 6 and 24 h. Data represent mean ± S.D., n = 3.

Table S1. Pharmacokinetic parameters of Rhein-DCA conjugate.

**Parameters**	T_{max} (h)	C_{max} (μg/mL)	$T_{1/2}$ (h)	$AUC_{(0-t)}$ (μg h/mL)	$AUC_{(0-\infty)}$ (μg h/mL)	CL (L/h/kg)	V_d (L/kg)
**I.G. (20 mg/kg)**	1.5 ± 0	2.68 ± 0.58	5.54 ± 3.38	8.22 ± 0.33	8.98 ± 1.21	2.25 ±0.29	17.11 ± 8.08

Data represent mean ± S.D., n = 3.
